# 27nt-RNAs guide histone variant deposition via ‘RNA-induced DNA replication interference’ and thus transmit parental genome partitioning in *Stylonychia*

**DOI:** 10.1186/s13072-018-0201-5

**Published:** 2018-06-12

**Authors:** Jan Postberg, Franziska Jönsson, Patrick Philipp Weil, Aneta Bulic, Stefan Andreas Juranek, Hans-Joachim Lipps

**Affiliations:** 1 Clinical Molecular Genetics and Epigenetics, Centre for Biomedical Education and Research (ZBAF), Faculty of Health, Witten/Herdecke University, Alfred-Herrhausen-Str. 50, 58448 Witten, Germany; 2 HELIOS University Hospital Wuppertal, Centre for Clinical and Translational Research (CCTR), HELIOS Medical Centre Wuppertal, Witten/Herdecke University, Heusnerstr. 40, 42283 Wuppertal, Germany; 3 Institute of Cell Biology, Centre for Biomedical Education and Research (ZBAF), Witten/Herdecke University, Witten, Germany; 4 iPSC CRISPR Facility, European Research Institute for the Biology of Ageing (ERIBA), University Medical Center Groningen, Groningen, The Netherlands

## Abstract

**Background:**

During sexual reproduction in the unicellular ciliate *Stylonychia* somatic macronuclei differentiate from germline micronuclei. Thereby, programmed sequence reduction takes place, leading to the elimination of > 95% of germline sequences, which priorly adopt heterochromatin structure via H3K27me3. Simultaneously, 27nt-ncRNAs become synthesized from parental transcripts and are bound by the Argonaute protein PIWI1.

**Results:**

These 27nt-ncRNAs cover sequences destined to the developing macronucleus and are thought to protect them from degradation. We provide evidence and propose that RNA/DNA base-pairing guides PIWI1/27nt-RNA complexes to complementary macronucleus-destined DNA target sequences, hence transiently causing locally stalled replication during polytene chromosome formation. This spatiotemporal delay enables the selective deposition of temporarily available histone H3.4K27me3 nucleosomes at all other sequences being continuously replicated, thus dictating their prospective heterochromatin structure before becoming developmentally eliminated. Concomitantly, 27nt-RNA-covered sites remain protected.

**Conclusions:**

We introduce the concept of ‘RNA-induced DNA replication interference’ and explain how the parental functional genome partition could become transmitted to the progeny.

**Electronic supplementary material:**

The online version of this article (10.1186/s13072-018-0201-5) contains supplementary material, which is available to authorized users.

## Background

Ciliated protozoa are single-celled eukaryotes that contain two types of nuclei—germline micronuclei and somatic macronuclei. A macronucleus develops from a micronucleus-derivative during sexual reproduction, starting when two cells of different mating types conjugate [1, 2]. Macronuclear development is associated with a programmed diminution of germline-specific DNA. The merits of two pioneering studies’ authors must be acknowledged since their fundamental observations suggested recently that in *Oxytricha* (class: Spirotrichea) small non-coding RNAs (sncRNAs) of 27nt in size are synthesized from the parental macronucleus. These target macronucleus-specific sequences in developing macronuclei in association with the PIWI homolog OTIWI-1. These authors proposed that the 27nt-RNAs protect specific sequences from being degraded [3, 4].

Being related to *Oxytricha*, *Stylonychia lemnae* is a spirotrichous ciliate species that has a long history as a model for macronuclear differentiation. Both species’ last common ancestor had probably lived approx. 500–650 myr ago [5]. In *Stylonychia*, developmental chromatin reorganization eventually leads to the formation of > 16,000 DNA fragments (‘nanochromosomes’) in the mature macronucleus of which most contain only one or few genes [6]. Priorly, over 95% of the micronuclear sequences become degraded. Most of these sequences comprise of repetitive and unique sequences from micronucleus-specific ‘bulk’ DNA [1]. Apart from this intergenic DNA, internal eliminated sequences (IESs) interrupt modules of macronucleus-destined sequences (MDSs) within many micronuclear genes. Frequently, MDS modules occur in a scrambled disorder, when compared with their proper arrangement in mature nanochromosomes [7]. The interrupting IESs must be removed during macronuclear differentiation. IESs can be as short as 10 bp [8]. Morphologically, prior to the developmental reduction of germline-specific sequences, the diploid zygote genome undergoes polytene chromosome formation in a phase of serial DNA replication steps [9]. At the onset of this stage IES excision and reordering of MDSs take place [10]. Thereafter follows the massive reduction of bulk DNA sequences, eventually leading to the breakdown of polytene chromosomes [1, 2]. Ultrastructural studies showed that excised DNA occurs in form of condensed chromatin [11]. In *Stylonychia*, the contribution of histone post-translational modifications (PTM) to the formation of heterochromatin was deeply studied [12–14]. Importantly, H3K27me3 becomes associated with micronucleus-specific sequences, and recent studies demonstrate that the deposition of histone variant-containing nucleosomes into chromatin of different nuclear types and their association with specific sequence classes play a role in developmental chromatin reorganization [12], suggesting that the spatiotemporal occurrence of the multiple histone variants in the life cycle of *Stylonychia* (2 linker histone H1 variants, 6 histone H2A variants, 4 H2B variants, 9 histone H3 variants and 2 histone H4 variants) is highly regulated. Moreover, observations that macronuclear development depends on sncRNAs [13] and the Argonaute-family protein PIWI1 in spirotrichs originate from *Stylonychia* studies, according to which PIWI1 could be a driver for RNA trafficking and transnuclear crosstalk [14, 15]. Evidence accumulates that another non-coding RNA species (‘template RNA’) might be involved in the reordering of MDS modules, IES excision, nanochromosome copy number determination and telomere addition [16–19].

Here, we exploited *Stylonychia* to perform a detailed dissection of developmental transcriptome patterns (27nt-RNA and mRNA). Further, we studied the enrichment of PIWI1, histone variants and PTMs with respects to the spatiotemporal course of programmed chromatin elimination. The integration of all these data allows us to propose that PIWI1/27nt-RNA complexes bind to complementary MDS sequences by RNA/DNA base-pairing. This could lead to a local delay in DNA replication during polytene chromosome formation. We assume that this delay could locally prevent the enrichment of H3.4K27me3, whose transient availability correlated with the critical time window of locally stalled DNA replication. In parallel, bulk DNA sequences not protected in this way could become differentially associated with H3.4K27me3 during their ongoing replication and thus being marked for elimination. Our in vitro and in vivo data support the idea that this ‘RNA-induced DNA replication interference’ (RIRI) could exist in vivo.

## Methods

### Cells

*Stylonychia* growth, conjugation and purification different nuclear types were described previously [1, 14].

### Nucleic acids labelling, probes and oligonucleotides

Nucleic acids labelling (5-fluorouridine [5FU] for nascent RNA and 5-iodo-2′-deoxyuridine [IdU] or 5-chloro-2′-deoxyuridine [CldU] for nascent DNA), in situ antibody stainings, poly[A]-RNA FISH and subsequent confocal microscopy were done as reported [20, 21]. A list of oligonucleotides used is provided as Supplemental Information (Additional file 1: Table S1).

### RNA isolation

Total RNA was isolated using Trizol (Sigma-Aldrich) and isopropanol precipitation and further purification on columns. Next, RNA integrity was assayed using the Agilent Bioanalyzer 2000. Only samples with non-fragmented RNA were included.

### DNA isolation

Genomic DNA was isolated from purified nuclei using phenol/chloroform/isoamylic alcohol extraction followed by ethanol precipitation.

### mRNA-seq and analyses pipeline

Whole transcriptome analyses were performed using total RNA. For a greater purity of mRNAs, we performed poly[A] purification using the NEBNext Poly(A) mRNA Magnetic Isolation Module (New England Biolabs), followed by transcriptomic library preparation (NEBNext Ultra RNA Library Prep Kit for Illumina). Seven libraries were multiplexed per lane and sequenced on a Illumina HiSeq 2000 platform (single end, 50 bp). This work has benefited from the facilities and expertise of the high throughput sequencing core facility of IMAGIF Gif-sur-Yvette (Centre de Recherche de Gif—www.imagif.cnrs.fr). The initial data analysis pipeline was as follows: CASAVA-1.8.2 was used for demultiplexing, Fastqc 0.10.1 for read quality assessment and Cutadapt-1.3 for adaptor trimming, resulting in sequence number for each developmental time point sample between 29.9 and 34.6 Mbp. File conversions, filtering and sorting as well as mapping (Bowtie2), were done using ‘Galaxy’ [22–24], a platform for data intensive biomedical research (https://usegalaxy.org/), or ‘Geneious 8.0′ software [25], respectively. Differentially expressed mRNAs could be identified after normalization using 18 calibrator genes (alpha tubulin, 40 s ribosomal protein S27, actin I [g4273], actin [g4210], GAPDH, glucose-6-phosphate isomerase, proteasome subunit beta [g9143], proteasome subunit beta [g15078], ER membrane protein complex subunit 3, charged multivesicular body protein 1a, vacuolar protein sorting-associated protein 26, ubiquitin C [g11902], ubiquitin C [g18215], nuclear cap-binding protein subunit 2, HSP90, HSP70, casein kinase 2 and eukaryotic translation initiation factor 3).

### Small RNA-seq and analyses pipeline

Total RNA was purified as described above. For multiplexing we made use of up to 10 different multiplex sequencing barcodes for sequencing in a single lane. This way, the entirety of the small RNAs could be analysed at a maximal level of time and cost efficiency. Total RNA was separated by polyacrylamide gel electrophoresis. Gel fragments corresponding to 15–35nt RNA molecules were cut, and RNA was eluted. These small RNAs were directly used for the construction of sequencing libraries in 4 steps. Step 1: ligation of DNA oligos to the 3′-end of the RNA; Step 2: ligation of RNA or, respectively, chimeric RNA/DNA oligos to the 5′-end of RNAs; Step 3: cDNA library synthesis by reverse transcriptase; Step 4: amplification of the cDNA library. Subsequently, after final quality checks by microcapillary electrophoresis and qPCR, the libraries were sequenced on an Illumina HiSeq 2000 platform (single end, 50 bp). Also here, this work has benefited from the facilities and expertise of the high throughput sequencing core facility of IMAGIF Gif-sur-Yvette (Centre de Recherche de Gif—www.imagif.cnrs.fr). The initial data analysis pipeline was as follows: CASAVA-1.8.2 was used for demultiplexing, Fastqc 0.10.1 for read quality assessment and Cutadapt-1.3 for adaptor trimming, resulting in an average sequence number for each developmental time point sample of 7.58 Mbp. File conversions, filtering and sorting as well as mapping (Bowtie2), were done using ‘Galaxy’ [22–24], a platform for data intensive biomedical research (https://usegalaxy.org/), or ‘Geneious 8.0′ software [25], respectively.

### RNA/DNA-IP-seq

Immunoprecipitation of RNA/DNA hybrids (RDIP) was done as described previously with slight modifications [26] and using the S9.6 monoclonal mouse antibody (Kerafast). This antibody was reported to bind DNA-RNA hybrids with high affinity, and its biophysical properties were determined in detail: According to the work of Phillips and co-workers [27], the S9.6 antibody exhibits dissociation constants of approximately 0.6 nM for DNA-RNA and some comparably weaker cross-reactivity of approx. 2.7 nM for RNA–RNA hybrids being AU-rich. The suitability of this antibody in IPs was further in demonstrated in several publications. Interestingly, a recent study demonstrates furthermore that the S9.6 antibody is suitable to detect RNA/DNA hybrids in R-loop structures in polytene chromosomes of *Drosophila* [28]. Moreover, several other published data demonstrate the high specificity of the S9.6 antibody (many listed on the manufacturer’s website: https://www.kerafast.com/product/1552/anti-dna-rna-hybrid-s96-antibody).

We found by direct comparison that RNA/DNA hybrids survive Trizol treatment in similar ways as the extraction with phenol/chloroform/isoamylic alcohol, despite the chaotropic effects of guanidinium. Briefly, following purification, whole nucleic acids were sonicated to a fragment length of approx. 400–600 bp and then treated with RNase I for ssRNA digestion. Nucleic acids were resolved in EB buffer (Qiagen, Hilden, Germany) after extraction with phenol/chloroform/isoamylic alcohol. For immunoprecipitation 3 µg nucleic acids per sample were incubated overnight with 2.5 µg anti-RNA/DNA hybrid [S9.6] mouse monoclonal antibody and 25 µL magnetic protein A beads (Diagenode). Following the purification of immunocomplexes and several washes with PBS, the samples were heated to 95 °C for 15 min, and then RNAs were purified using Trizol. Small RNA libraries for massive parallel sequencing were made as described above.

### Microscopy

Immunodetection of PIWI1 and H3K27me3 was done as described [12, 14].

### RNA interference

For PIWI1-RNAi *Stylonychia* were treated as outlined [12, 14].

### TtAgo expression

For TtAgo expression we made use of the same plasmids for TtAgo expression, which have been made available by those authors (Addgene), i.e. pWUR702 (TtAgo) and pWUR703 (loss-of-function TtAgo [D478A, D546A]). The expression and purification were performed as described [29].

### PIWI1 expression, PAR-CLIP on wild-type PIWI1 and pull-down

For cell-free reticulocyte lysate-based in vitro expression (TNT Quick System, Promega) of PIWI1, we cloned the gene with N-terminal His-tags into pCMV-TNT (Promega). Priorly, we ordered synthetic versions of each gene (GenScript) to ‘universalize’ the deviant genetic code used by *Stylonychia*, where TAA and TAG encode for glutamine instead of a termination signal. For enrichment of His-PIWI1, we utilized Ni–NTA Magnetic Agarose Beads (Qiagen) upon manufacturers recommendation.

We performed photoactivatable-ribonucleoside-enhanced cross-linking and immunoprecipitation (PAR-CLIP) to confirm that PIWI1 binds to the 27nt-RNAs. Therefore, we pulled down PIWI1 after 4-thiouridine (4TU)-labelling of nascent RNA using a 1:100 dilution (1 mM) from a 1 M 4TU stock solution (in DMSO) in exconjugants approx. 18 h PC incubated for 2 h. Subsequent cross-linking of *Stylonychia* was performed on a gauze with UV light (365 nm). Enriched PIWI1-RNPs were end-labelled using polynucleotide kinase and ^32^P followed by denaturing SDS-PAGE. X-ray film detection was used for PIWI1 protein band visualization. For further characterization RNA-seq and coverage studies on small RNAs purified from pulled-down PIWI1 immunocomplexes were performed. For native PIWI1 precipitation in combination with PAR-CLIP or as a standalone experiment, we used the polyclonal rabbit anti-PIWIL1 antibody (Abcam, #ab12337), which was previously shown to bind *Stylonychia* PIWI1/mdp1 [14] and *Oxytricha* Otiwi1 [3]. For immunoprecipitation chromatin fractions from purified macronuclear anlagen were incubated overnight with 3 µg anti-PIWIL1 and 25 µL magnetic protein A beads (Diagenode). Following the purification of immunocomplexes and several washes with PBS, the samples were heated to 95 °C for 15 min, and then RNAs were purified using Trizol. Small RNA libraries for massive parallel sequencing were made as described above.

### PCR

End-point PCR was performed to analyse the processing of IES excision and bulk DNA elimination and to study the effects of 27nt-RNAs on the efficiency of *Pfu* and *Taq* polymerase.

Quantitative real-time PCR was performed to monitor the amplification of reporter amplicons during chromosome polytenization and to study the effects of Argonaute/oligonucleotide complexes on the amplification of a PCNA amplicon. Oligonucleotides used as primers or for interference experiments are listed in Additional file 1: Table 1; primers were used in a 0.5 µM concentration.

Using the PCNA-template assay system and *Taq* polymerase as described above for qPCR, we replaced 27nt-RNA with 5′-phosphorylated 21nt-DNA.

End-point PCR was carried out on a VWR Doppio PCR device using Maxima Hot Start *Taq* Polymerase (ThermoFisher) or *Pfu* DNA Polymerase (ThermoFisher). Standard PCR conditions were as follows: 95 °C for 10 min, 30 cycles of [95 °C for 15 s, 60 °C for 30 s, 72 °C for 30 s], 72 °C for 10 min; alternatively, for the 856 bp PCNA amplicon the conditions were: 95 °C for 15 min, 30 cycles of [95 °C for 15 s, 60 °C for 30 s, 72 °C for 45 s], 72 °C for 10 min.

Quantitative PCR was performed on a Rotor Gene 6000 (Corbett Life Science) using QuantiTect SYBR green *Taq* polymerase Master Mix (Qiagen) or *Pfu* DNA polymerase (ThermoFisher) and SYBR green (Sigma-Aldrich). Standard PCR conditions were as follows: 95 °C for 15 min, 40 cycles of [95 °C for 15 s, 60 °C for 30 s]; alternatively, for the 856 bp PCNA amplicon the conditions were: 95 °C for 15 min, 40 cycles of [95 °C for 15 s, 60 °C for 45 s]. Melting of PCR product was performed using a temperature gradient from 55 °C to 95 °C, rising in 0.5 °C increments.

### Klenow reaction

To analyse the effect of PIWI1/27nt-RNA in a linear DNA amplification reaction at ambient temperature, we performed a *Klenow* reaction using the large fragment of DNA polymerase (ThermoFisher) and pGEM-T-easy-PCNA as template upon manufacturers’ recommendations. For priming a modified T7 sequencing primer was used (Additional file 1: Table S1). The amount of polymerized PCNA sequence was successively analysed using qPCR as described above.

### Microinjection of 27nt-RNA

Injection of single-stranded 27nt-RNA (Additional file 1: Table S1) in synchronized vegetative cells before the onset of replication was performed as described before [30]. Injection time of 1.0 s and three injection pulses per cells were applied leading to microinjection of about 3–5 pL per cell. RNA was injected into the cytoplasm near to the macronucleus. We estimated the copy numbers of targeted nanochromosomes using know copy numbers [17] and read coverage data from the macronucleus genome [6] for normalization. 27nt-RNAs were diluted in Pringsheim solution [1] to achieve an approx. 100-fold excess over the nanochromosome copy numbers. Copy number changes were assayed using qPCR as described above with primers resulting in short amplicon from H3.7 (57 bp), MDP2 (97) or PCNA1 (103 bp) (Additional file 1: Table S1).

## Results

### An cornucopia of histone variant genes and RNA metabolism genes dominate the transcriptomic orchestra during sexual reproduction

**Fig. 1:**
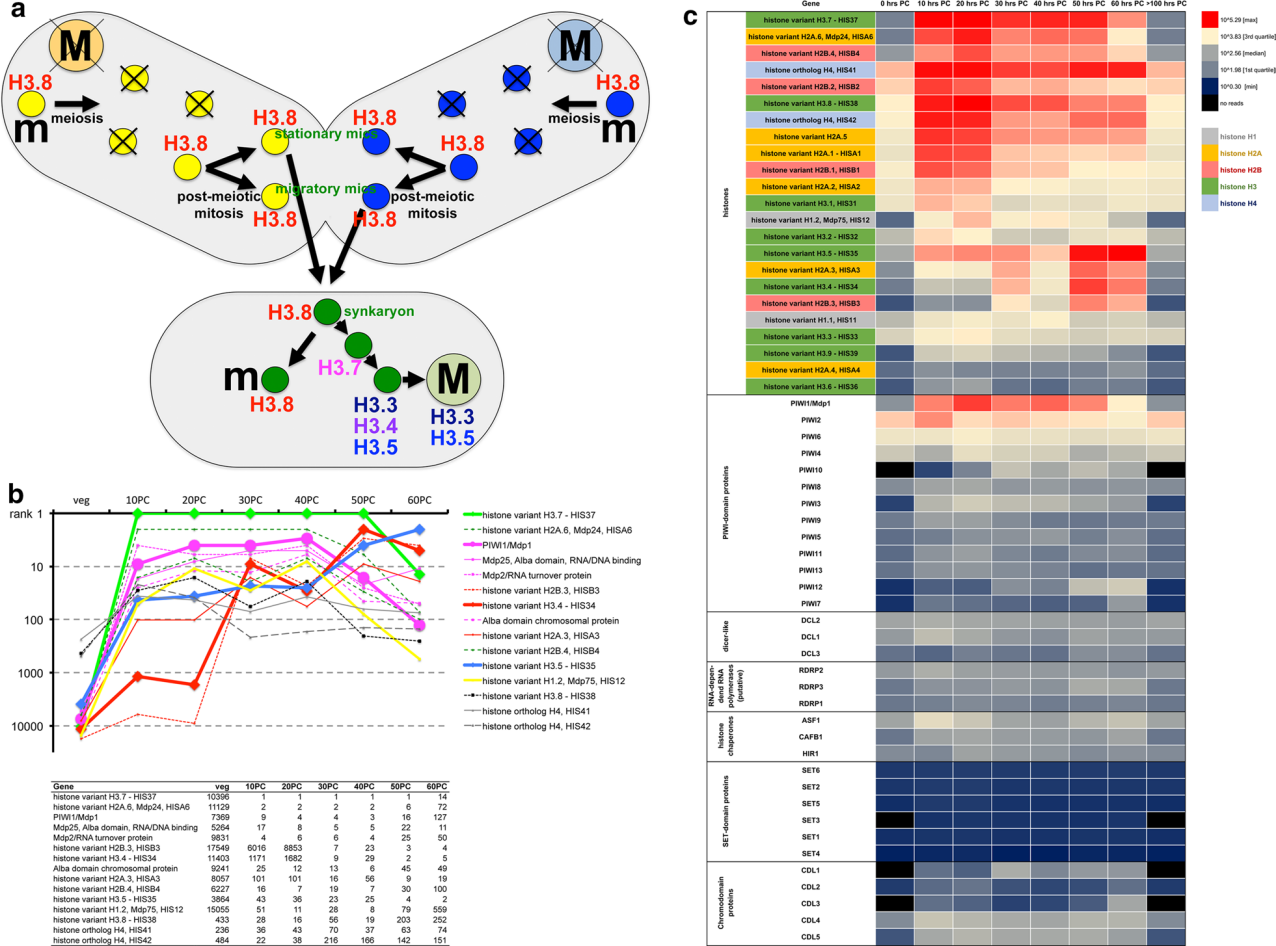
Spatiotemporal dynamics of histone variants in *Stylonychia* and top-ranked differentially expressed genes during macronuclear development. **a** H3.8 is a micronucleus-specific H3 variant that possesses a specific PTM signature (H3.8K33me3T34ph; homolog to H3K27me3S28ph) and becomes replaced by another variant (H3.7) in developing macronuclei. Subsequently H3.7 also becomes replaced by H3.3, H3.4 and H3.5. **b** Ranking diagram (top) and table (bottom) demonstrating the differential expression of selected gene transcripts. Many histone variant genes and RNA metabolism genes are under the top ranking genes, which become differentially expressed during macronuclear development. **c** Genes of interest being differentially expressed during macronuclear development (10 to > 100 h PC) were identified by their relative abundance and expression fold change in comparison to vegetative (v) *Stylonychia.* Explicitly, among the histone genes several variants are abundantly expressed during development: H2A.6 (early polytenization/EP) and H2A.3 (late polytenization/LP), H2B.4 (EP) and H2B.3 (LP), H3.7 (EP), H3.5 (EP/LP), H3.4 (LP). Also the turnover of histone H4 (EP/LP), the linker histone variant H1.2 (EP/LP) as well as the histone chaperone ASF1 is markedly elevated. PIWI1 (also macronuclear development protein1; MDP1) is by far the most abundant PIWI-domain protein expressed during macronuclear development, whereas PIWI2 and much lower PIWI6 become constitutively expressed. 10 other PIWI-like genes remain comparatively lowly expressed. Besides, three DICER candidate genes (DCL1-3), three putative RNA-dependent RNA polymerases (RDRP1-3) exhibit development-specific escalation of transcription on a low level. A somehow unexpected paucity of putative chromatin modifier genes (chromodomain proteins [CDL1-5] and putative SET-domain KMTs [SET1-5]) was moreover observed

A unifying mechanistic model for developmental sequence protection through sncRNAs integrating the many known factors and mechanisms, which contribute to programmed chromatin reorganization is lacking to date for spirotrichs like *Stylonychia* and *Oxytricha*. For the purpose of identifying important differentially expressed genes and to connect important gene networks, we performed deep sequencing of mRNA sampled at 8 stages in the sexual cycle of *Stylonychia* (i.e. vegetative cells, cells at the onset of conjugation (0 h post-conjugation [PC]) and after consecutive intervals 10–60 h PC). With emphasis on the probable important roles of histone variants in the course of macronuclear development, Fig. 1a summarizes the current knowledge on the spatiotemporal localization of several histone H3 variants. Our mRNA-seq results now underlined the superior roles of both histone variants and sncRNA metabolism factors in macronuclear differentiation, since many genes involved were ranked under the top differentially expressed candidates (Fig. 1b). Remarkably, during the same corresponding stages of macronuclear differentiation in *Oxytricha trifallax*, disproportionate numbers of mRNAs putatively encoding proteins involved in RNA (and DNA) functions were described recently [31]. Somewhat unexpectedly, we noted a paucity of chromodomain proteins and putative histone KMTs with only few candidates being developmentally expressed (Fig. 1c). This seemed to be a major difference to *Tetrahymena*, where such proteins are centrally for developmental DNA elimination [32, 33]. Notably, in contrast to the many *Stylonychia* histone variants, we could identify a uniform set of histone chaperones only, i.e. at a time one homolog of mammalian ASF1, CAF-1 and HIRA, respectively.

### 27nt-RNAs occur during development, whereas 21–22nt-RNAs become constitutively synthesized

**Fig. 2:**
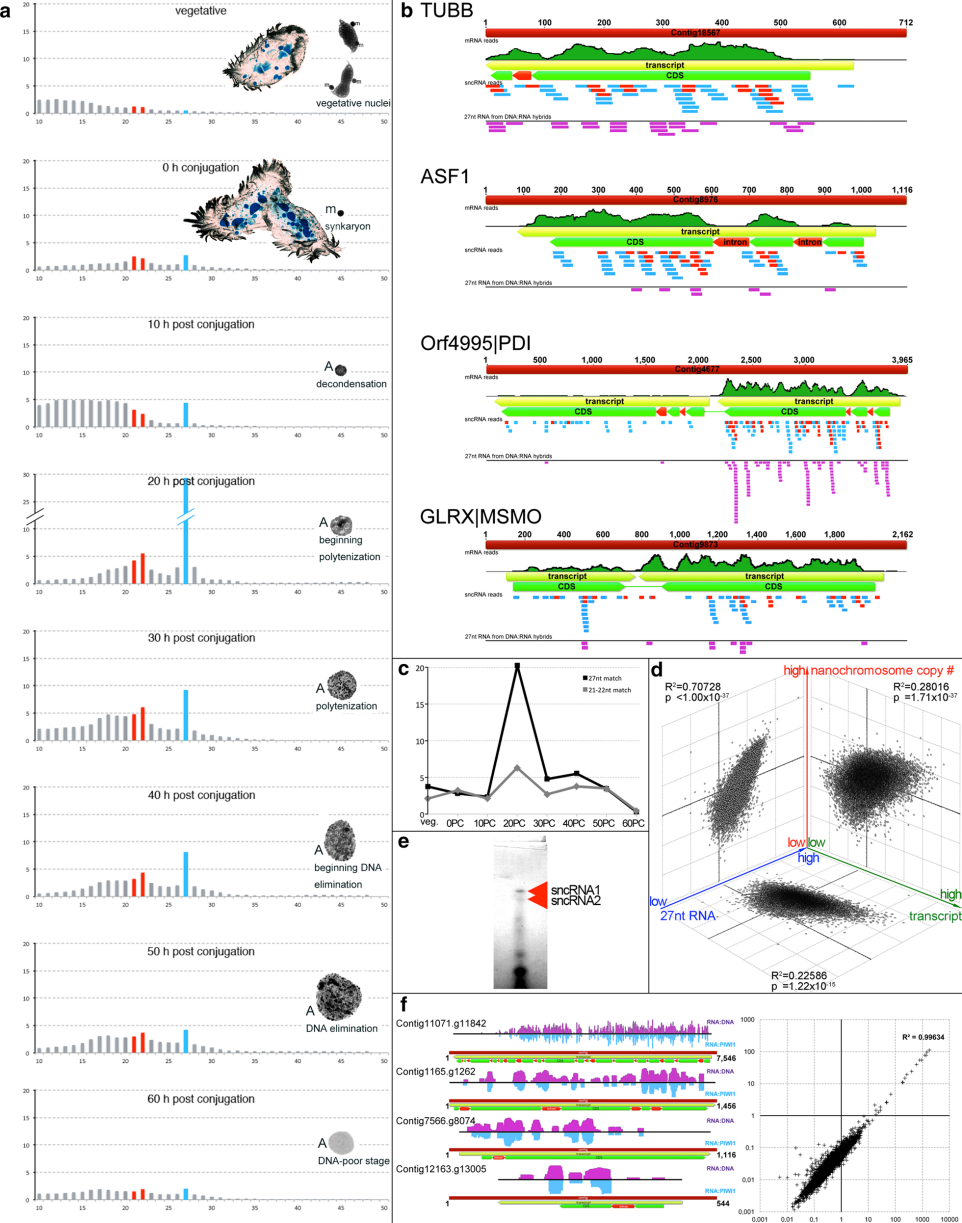
Characterization of 27nt-RNAs and their association with macronuclear sequences. **a** Time course of small RNA fragment size distribution (10–50nt) and associated stages of macronuclear development showing the dominance of 27nt—RNAs (light blue columns). Red columns mark 21–22nt-RNAs. The y-axis shows the normalized read quantity (× 10^5^). **b** The illustration shows the coverage of 27nt-RNAs (light blue bars), 21–22nt-RNAs (red bars) and mRNA reads (green curves) on four exemplary nanochromosomes with complete telomere-to-telomere sequence (red contigs). Notably, the mapped area frequently includes introns (1.95% of 27nt-RNA/1.43% of 21–22nt-RNA). The position of predicted full-length transcripts (yellow arrows) and coding sequences (CDS; green arrows, occasionally interrupted by introns [red arrows]) is illustrated. The bars below (purple) show furthermore the coverage of 27nt-RNAs, which were purified from pulled-down RNA/DNA hybrids using the mouse anti-RNA/DNA hybrid [S9.6] monoclonal antibody (mAb). **c** Average sncRNA (27nt-RNAs and 21–22nt-RNAs) read counts matching per contig. **d** Combined correlation analyses between mRNA reads (transcript), 27nt-RNAs and the relative nanochromosome copy numbers. **e** PAR-CLIP revealed a prominent RNA band well in agreement with the expected size of a PIWI1/27nt-RNA complex (sncRNA1)), whereas a smaller, at best very faint band could correspond to the expected size of a PIWI1/21–22nt-RNA complex (sncRNA2). **f** The read coverage is shown on the left for 4 exemplary nanochromosomes for 27nt-RNAs enriched in immunocomplexes using the mouse anti-RNA/DNA hybrid [S9.6] mAb (Kerafast #ENH001) (purple signals) or, respectively, rabbit anti-PIWIL1 polyclonal antibody (pAb) (Abcam #ab12337) (light blue signals). Symbolism and colour coding of the nanochromosome annotation equals (**b**). The chart (right) shows the results of correlation analyses between the reads obtained from 27nt-RNA-seq (*x*-axis: RNA/DNA-IP; *y*-axis: PIWI1-IP)

Deep sequencing of small RNAs purified from the same samples as described above revealed two different size fractions becoming developmentally enriched, which were 21–22nt and 27nt in size, respectively, reminiscent of *Oxytricha* [3, 4]. We identified 21–22nt-RNAs at very low levels over threshold in isolates from vegetative cells and in the course of development (Fig. 2a). Although these small RNAs were almost undistinguishable from similarly sized fragments, their 5′- and 3′-bias as well as the internal base composition was reminiscent of 27nt-RNAs (Additional file 1: Figure S1A). This conservation appeared to rule out that the 21–22nt-RNAs were simple decay products.

In contrast, while an occurrence of 27nt-RNAs over baseline was not detected during vegetative growth, they emerged at the onset of conjugation and were clearly visible above threshold 10 h PC—at a time point, when the chromatin topology of prospective macronuclei (anlage[n]) exhibited a less condensed state when compared with micronuclei. Thereafter, we observed a massive 27nt-RNA gain (approx. 20 h PC, at the onset of polytene chromosome formation). Subsequently, 27nt-RNAs were still enriched until 60 h PC. At this time point, macronuclear anlagen entered a DNA-poor stage, which followed bulk DNA elimination. After rapidly reaching their maximum at ~ 20 h PC, the concentration of the 27nt-RNAs decreased continuously. Notably, the occurrence of other small-sized RNA (< 21nt) fragments overlapped with the synthesis of 27nt-RNAs, possibly representing debris of the sncRNA biogenesis.

### Both 21–22nt-RNAs and 27nt-RNAs map exclusively to transcribed macronuclear sequences

We mapped both sncRNA fractions to the macronuclear genome and to all known micronuclear sequences (complete model genes and thousands of preliminary micronuclear contigs). Clearly and reminiscent of *Oxytricha* [3, 4], the vast majority of 21–22nt-RNAs (86.46%) and almost all 27nt-RNAs (98.04%) covered the transcribed regions of nanochromosomes at 20 h PC. Due to their greater abundance 27nt-RNAs had a much higher coverage than 21–22nt RNAs. Figure 2b shows exemplarily how both sncRNA fractions and mRNA reads mapped to four nanochromosomes—two containing single genes (TUBB and ASF1) and two dual gene nanochromosomes (Orf4995|PDI and GLRX|MSMO). Notably, the non-transcribed subtelomeric regions of the nanochromosomes were omitted from sncRNA read coverage, indicating that not the entire body of MDSs in the developing macronucleus became targeted by 27nt-RNAs—an interesting point we will take up below in the discussion.

We did not observe mapping of 21–22nt/27nt-RNA to micronucleus-specific genomic sequences. Almost all reads not covering MDSs could be assigned to other sources, e.g. sequences of ribosomal or mitochondrial origin, mRNA or repetitive sequences from ingested *Chlorogonium* algae (food source for *Stylonychia*), plastids or bacteria (Additional file 1: Figure S1B). Notably, these results challenge earlier observations in *Stylonychia*, where a small RNA probe preferentially appeared to hybridize to micronucleus DNA instead of macronucleus DNA in Southern analyses [13]. We assume that these earlier results were false positive. This could possibly be caused by probe purity and/or specificity issues, e.g. very short probe molecules (therefore less specific) were hybridized to micronuclear DNA, which is rich in repetitive sequences, at intermediate hybridization temperatures of 40 °C. Moreover, a relatively early sampling time point was selected in that previous study (10 h PC). Thus, enrichment of 27nt-RNAs was presumably not at its peak and the probe presumably contained other small nucleic acid contamination not originating from MDSs (compare Fig. 2a, c). We thus expect that the ratio between MDS-specific 27nt-RNAs and unspecified RNA fragments, which accumulated 10 h PC (Fig. 2a), led to an adverse probe composition.

Interestingly, the 27nt-RNAs coverage seemed to correlate directly with the mRNA copy number (*R*^2^ = 0.22586; *p *= 1.22 × 10^−15^) (Fig. 2b, d). This direct correlation left open the possibility that the substrate for the biogenesis of 27nt-RNAs could be pre-mRNA or mRNA. Supportive to this speculation was also that the 27nt-RNA reads covered introns to some extent when mapped to nanochromosomes, but not the non-transcribed subtelomeric regions. Thus, the 27nt-RNA read coverage overlapped well with the transcribed regions of the nanochromosomes.

Further, we tested whether the relative nanochromosomes copy numbers correlated with the 27nt-RNA coverage, since it is known that the mRNA copy numbers are influenced by the individual nanochromosome copy numbers [2, 17], whereby the different expression levels of genes residing on dual gene nanochromosomes show that the nanochromosome copy number is not the only determinant for mRNA quantity (Fig. 2b; Orf4995|PDI, GLRX|MSMO). However, we found a high positive correlation of both mRNA and nanochromosome copy numbers (*R*^2^ = 0.70728; *p *< 1.00 × 10^−37^) (Fig. 2d).

In addition, we observed that the mapped read count of 27nt-RNAs increased massively, transiently and selectively about 20 h PC (Fig. 2c), while the sequence composition of the 27nt-RNA pool remained stable from the onset of conjugation and in the course of successive development (Additional file 1: Figure S1C, D). This was reminiscent of a selective 27nt-RNA amplification by RNA-dependent RNA polymerase activity, possibly by one of the identified RDRP candidates (Fig. 1c).

In sum, 27nt-RNAs obviously occupied an elevated functional position also for *Stylonychia*. Sequence homology suggested that 27nt-RNAs target MDSs in developing macronuclei, whereby the omission of non-transcribed nanochromosomal sequences that are contained in MDSs suggested that this targeting might be incomplete.

In the subsequent part we focussed on 27nt-RNA and decided to neglect 21–22nt-RNA for further analyses.

### 27nt-RNAs bound by PIWI1 hybridize to complementary MDSs in developing macronuclei

To gain deeper insight into the putative 27nt-RNA-mediated protection mechanism for MDS retention, we studied the interactions of 27nt-RNAs with PIWI1 and with their target sequences, respectively. For this purpose, we purified macronuclear anlagen from *Stylonychia*, most of them being between 20 h and 40 h PC (beginning polytenization to beginning DNA elimination). These samples were allocated to different experiments.

To confirm that PIWI1 binds to sncRNAs, we performed PAR-CLIP. Therefore, we pulled down PIWI1 after 4TU-labelling of nascent RNA in exconjugants and cross-linking with UV light (365 nm). Enriched PIWI1-RNPs were end-labelled using polynucleotide kinase and ^32^P followed by denaturing SDS-PAGE. X-ray film detection of the radioactive tracer revealed a prominent signal being associated with the PIWI1 protein band (sncRNA1 in Fig. 2e) and another very faint smaller sized signal (sncRNA2 in Fig. 2e). Both signals were well in agreement with the expected molecular weight of PIWI1 loaded with 27nt-RNA (~ 98 kDa) or 21–22nt-RNA (~ 96 kDa), respectively, whereas higher molecular weight RNA was not present. The signal strength difference and the relative overrepresentation of the 27nt-RNAs over 21–22nt-RNAs suggested that PIWI1 binds predominantly 27nt-RNAs.

For further characterization RNA-seq and coverage studies on co-purified sncRNAs from pulled-down PIWI1 immunocomplexes were performed. For threshold subtraction and normalization, we used isotype control antibodies not specific for PIWI1. Taken together, we yielded substantial enrichment of 27nt-RNAs associated with pulled-down PIWI1. Deep sequencing revealed a coverage pattern on transcribed sequences of nanochromosomes, which was well in accordance with the data obtained for RNA-seq on purified small RNAs from serial stages of macronuclear development (Fig. 2f). Next, we intended to study the nature of MDS targeting by 27nt-RNAs, i.e. in particular, whether 27nt-RNAs bind directly to their target sequences via base-pairing: We purified nucleic acids from the same samples as described above and used the mouse monoclonal S9.6 antibody for immunoprecipitation of RNA/DNA hybrids [26].Again, negative isotype controls were performed. Subsequently, we made use of deep sequencing to analyse the coverage of 27nt-RNAs contained in these immunocomplexes. Strikingly, we observed a very high level of congruency between the coverages of 27nt-RNAs purified from RNA/DNA hybrids and previously purified sncRNAs at 20 h PC (Fig. 2b) as well as the coverage of 27nt-RNAs purified from pulled-down PIWI1 complexes (Fig. 2f). Here, *R*^2^ was as high as 0.99634 (Fig. 2f). In summary, these observations suggested that 27nt-RNAs are bound by PIWI1. The resulting PIWI1/27nt-RNA complexes could function as guides to target complementary sequences in MDSs by RNA/DNA base-pairing.

### Intergenic bulk DNA elimination, but not IES removal depends on PIWI1

Although the above results revealed the deepest characterization of 27nt-RNAs bound to PIWI1 in *Stylonychia* so far, earlier studies already suggested that the sncRNA turnover is associated with PIWI1 [12, 14, 15]. By RNA-seq we now confirmed and complemented older results from microarrays [34], that PIWI1 was by far the most abundantly expressed developmental variant out of 13 PIWI-like proteins encoded in *Stylonychia* (Fig. 1). However, although we recently obtained evidence that PIWI1 influences the expression of some histone variants during macronuclear development [12], its mechanistic involvement is not yet understood. The quasi inverse sncRNA coverage pattern observed in *Oxytricha* as well as in *Stylonychia*—when compared with the ciliate *Tetrahymena*—could give support to the idea that alternative mechanisms could had evolved in the spirotrichous ciliate taxon, which fundamentally differ from *Tetrahymena*, where small non-coding ‘scan’ RNAs and the PIWI-homolog Twi1p specify micronuclear-limited sequences for heterochromatin formation [35]. Here, prior to chromatin elimination, heterochromatin formation involves H3K27me3 and H3K9me3 introduced by the histone lysine methyltransferase (KMT) EZL1 and binding of the chromodomain protein Pdd1p [32, 33]. This concept historically fed earlier speculations that the PIWI-domain proteins could directly interact with chromatin modifying factors also in spirotrichs. We now note that this view is challenged by the observed differences in sncRNA biology between these protozoa, and this impression is confirmed by the observed paucity of promising candidates for chromatin modifiers encoded in the *Stylonychia* genome, which would theoretically be required.

**Fig. 3:**
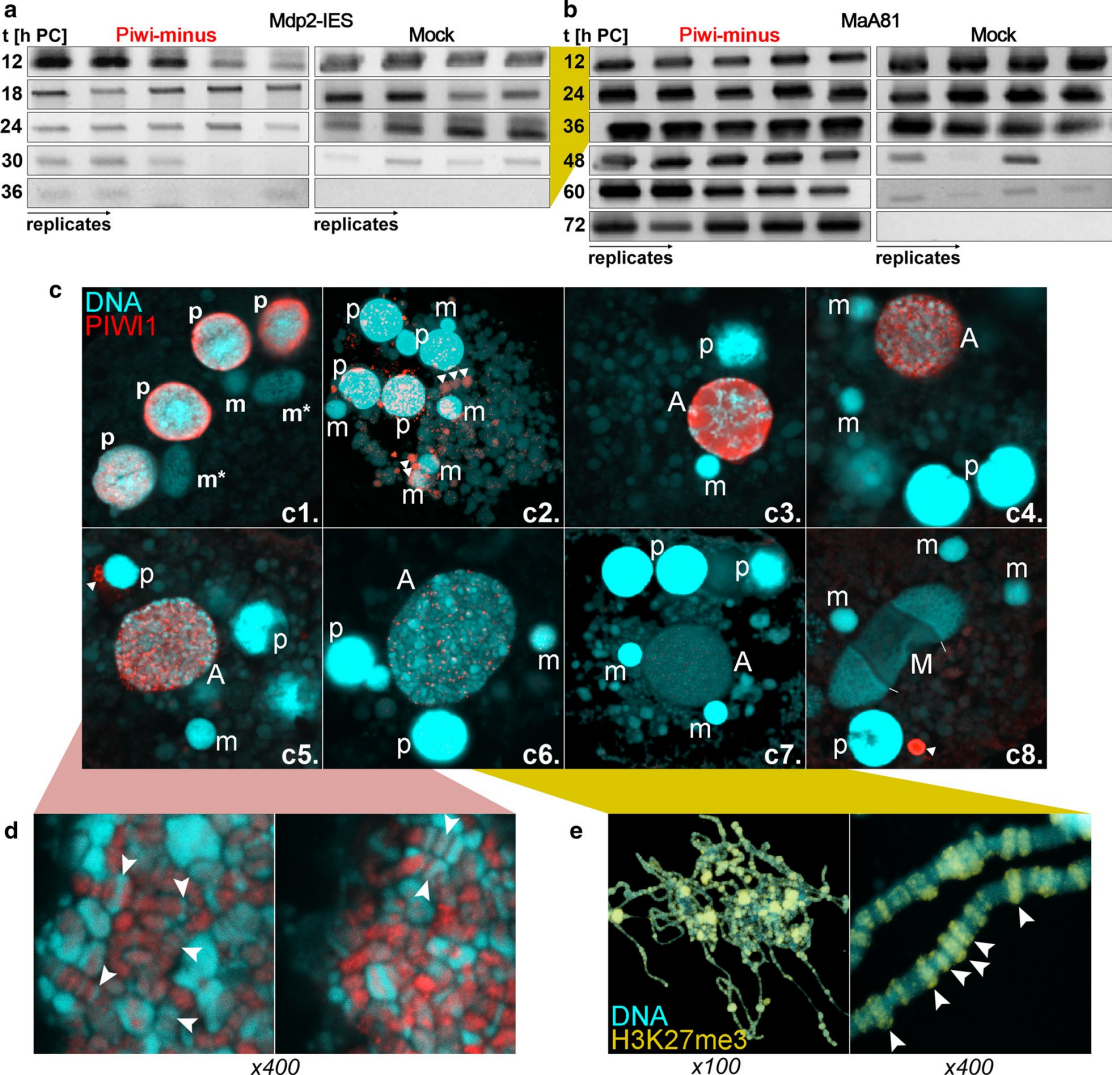
Differential excision of micronuclear sequence classes and immunolocalization of PIWI1 and H3K27me3 in polytene developing macronuclei. **a**, **b** Agarose gel electrophoresis of amplicons from PCR analyses used for the examination of sequence excision. x-axis/line labelling: time PC (hours). In each box the different columns represent replicate experiments. **a** MDP2 gene—PIWI1-minus samples (left) and non-RNAi control samples (right); **b** MaA81 repetitive bulk DNA—PIWI1-minus samples (left) and non-RNAi control samples (right). For each experiment several replicates are shown in columns. **c–e** Immunolocalization of PIWI1 (**c**, **d**; red) or H3K27me3 (**e**; yellow) during development (**c1-4**) and in polytene chromosomes at higher magnification (**d**, **e**). To-Pro-3 was used for DNA counterstaining (blue). Briefly as previously described [14], PIWI1 accumulates in parental macronuclei (p) during conjugation (**c1**) and persists immediately after cell separation (**c2**). Subsequently, PIWI1 vanishes from parental macronuclei and translocates to the prospective macronucleus (A) (**c3-7**). Thereafter, premature macronuclei, whose nanochromosomes undergo a second series of DNA replication until they reach their final copy numbers, become devoid of PIWI1. Remarkably, we observed an extranuclear PIWI1 spot and speculate that it could be a degradation body (**c8**). p: parental (old) macronucleus fragments; m(*): micronucleus (*during mitosis); A: anlage, developing macronucleus; M: macronucleus. Lines in c8 point to replication bands, the arrowhead points to an unknown PIWI1-positive extranuclear structure. Arrowheads in D point to areas, where the shape of the polytene chromosomes as well as the banding pattern can be well recognized

As a progressive step towards elucidating the mechanistic involvement of PIWI-domain proteins in spirotrichs, we continued to dissect the function of PIWI1. We used RNAi to study the effects of PIWI1 knock-down on the elimination of two different micronucleus-specific sequence classes—IESs and bulk DNA, since it was unknown to date whether the elimination of both depends on PIWI1 or not. Using PCR primer pairs amplifying only IES-containing micronucleus-specific sequences of selected genes, we observed that IES elimination from the macronucleus development protein 2 (Mdp2)-gene in PIWI1-minus samples was almost indistinguishable from controls—most IESs were removed between 24  and 30 h PC (Fig. 3a, Additional file 1: Figure S2). Contrary, a reporter sequence from the repetitive bulk DNA element MaA81 that is present in ~ 5000–7000 copies per haploid micronucleus genome [36] was almost completely eliminated 48 h PC in controls, whereas no DNA diminution was seen during prolonged observation periods up to 72 h PC in PIWI1-minus samples (Fig. 3b, Additional file 1: Figure S2). These results indicate that bulk DNA elimination, but not IES removal depended on PIWI1.

### PIWI1 and H3K27me3 exhibit mutually exclusive localization patterns in polytene chromosomes

To complement the hypothesis that 27nt-RNAs in association with PIWI1 supposedly protect sequences that must be retained during macronuclear development [3], we reinvestigated the nuclear localization of PIWI1 and H3K27me3 with improved microscopic resolution (see visualization of an intact macronuclear anlagen nucleus with polytene chromosomes in Additional file 1: Figure S3A and Fig. 3c_1-8_, d, e). A first time observed feature was that PIWI1 appeared to travel physically within extranuclear spheres from parental macronuclei to the young anlage, which at this time point morphologically could not yet be distinguished from micronuclei (m) (Fig. 3c_2_). In particular, extranuclear PIWI1 overlapped with faint To-Pro-3-positive spheres, indicating that they might have contained significant RNA concentrations. Improved resolution elucidated that PIWI1 subsequently swamped the young anlage, and only in later anlagen stages subtle PIWI1 foci shaped out (Fig. 3c_3-7_). Subsequently, these foci became gradually fewer in number. These patterns suggested that PIWI1/27nt-RNA complexes were specifically targeted to discrete sites. Strikingly, this suggestion became obvious when higher magnifications of polytene chromosomes were examined (Fig. 3d). Here, condensed chromatin bands were devoid of PIWI1, whereas PIWI1 signals traced the longitudinal shape of the polytene chromosomes in areas not associated with dense chromatin. This suggested that PIWI1 colocalized with uncondensed chromatin.

Complementary, H3K27me3 was selected as a marker for condensed chromatin (Fig. 3c–e). Inversely, when compared with PIWI1 patterns, solely the condensed chromatin bands were strongly stained when antibodies against H3K27me3 were used for immunofluorescence microscopy on polytene chromosomes (Fig. 3e).

Notably, with respect to a potential H3K27me3/K9me3 ambiguity (compare epitopes in Additional file 1: Figure S3B), we observed non-identical H3K27me3/H3K9me3 staining patterns demonstrating the discriminability of both PTMs by the antibodies in use (Additional file 1: Figure S3C): In contrast to H3K27me3, H3K9me3 did not occur in condensed chromatin bands of polytene chromosomes. During DNA elimination H3K9me3 occurred only within chromatin bodies that were pinched off the polytene chromosomes. Within those amorphous bodies also H3K27me3 and H3K9ac occurred. These bodies were reminiscent of ‘swollen compartments’ observed earlier by transmission electron microscopy in anlagen after polytene chromosome breakdown, which were interpreted to contain material from former individual bands of a polytene chromosome [37]. All these observations supported the idea that PIWI1/27ntRNA complexes covered MDSs, possibly to protect them against the formation of condensed chromatin by means of H3K27me3.

In addition to the key observation of alternating PIWI1/H3K27me3 patterns in polytene chromosomes, we noticed that prior to its enrichment in macronuclear anlagen cytoplasmic H3K27me3 accumulations occurred, which did not overlap with any type of nuclei (Additional file 1: Figure S3D-L). This discovery suggested that trimethylation of H3K27 took place before it was assembled into polytene chromosomes. Consequently, since a pool of cytoplasmic H3K27me3 appeared to exist prior to its enrichment in anlagen chromatin, a putative nuclear H3K27-specific histone KMT activity that selectively targets micronucleus-specific sequence-associated nucleosomes would not be required.

Considering all these data we developed the model that sequence protection could mechanistically be achieved, if 27nt-RNAs together with PIWI1 could cause an ‘RNA-induced DNA replication interference’—i.e. if 27nt-RNAs would impair DNA replication at covered MDS loci during polytenization, thus locally and temporarily reducing the de novo deposition of nucleosomes. If in the meantime one or more specific histone variants and/or specific H3K27me3 activity would be transiently enriched, heterochromatin formation should be limited to those regions not protected by 27nt-RNAs. Our earlier work suggested that the late introduction of H3K27me3 during polytenization might correlate with the best time window for nucleosome deposition into chromatin, which opens during DNA replication—here during polytenization [14]. We hypothesize in particular that meanwhile MDSs are protected through stalled replication, the transient availability of H3K27me3 would lead to its preferential enrichment in bulk DNA regions.

### DNA replication does not take place simultaneously at all sites during polytene chromosome formation in vivo

**Fig. 4:**
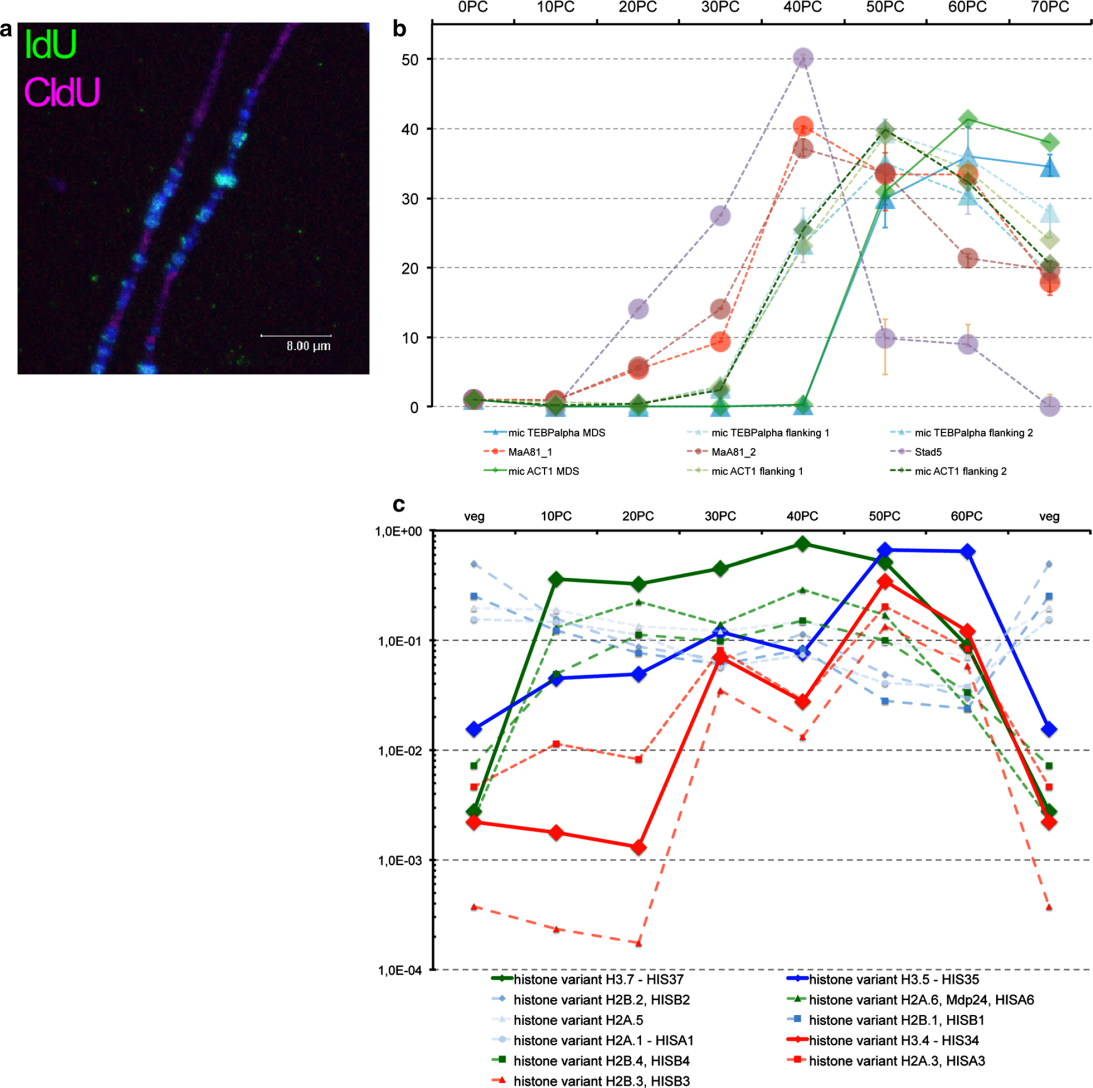
Polytenization does not takes place synchronously at all sites. **a** Immunolocalization of newly replicated DNA (1st pulse IdU [green], 2nd pulse CldU [red] in polytene chromosomes. To-Pro-3 was used for DNA counterstaining (blue). **b** Relative quantification of micronuclear gene sections in the course of macronuclear development by qPCR. **c** Quantification of histone variant transcripts of interest through internal normalization of each developmental time point to the histone H4 read count and normalization of each developmental time point to the read count of 15 housekeeping genes in vegetative cells. The y-axis represents the number of histone variant of interest per histone H4

Accordingly, during developmental chromosome polytenization DNA replication should not occur simultaneously at all sites. In fact, we recognized spatiotemporally regulated replication patterns in the micro- and macronuclei of vegetative *Stylonychia* previously [20]. To test whether differentially replicated regions exist also in polytene chromosomes, we performed pulse (5 h)–chase (1 h)–pulse (5 h) labelling of newly replicated DNA using the nucleotide analogues iododeoxyuridine (IdU; early pulse) and chlorodeoxyuridine (CldU; late pulse). Subsequently, labelled nascent DNA in anlagen or spread polytene chromosomes was analysed by immunofluorescence microscopy. We observed that IdU (green, 1st pulse) and CldU (red, 2nd pulse) occurred mostly separated from each other, with IdU being tentatively associated with condensed bands and CldU with less condensed chromatin areas (Fig. 4a). These observations suggested that non-synchronous replication occurred during polytene chromosome formation. To confirm, we used qPCR to examine a time course during polytenization using reporter amplicons from bulk DNA (MaA81 and Stad5, respectively) and two micronuclear model genes, whose MDS/IES architecture and flanking sequences are well characterized (telomere binding protein alpha [TEBPalpha] and actin I [ACT1]). We found that MaA81 and Stad5 started replication earlier than MDSs (Fig. 4b). Sequences, which directly flanked micronuclear genes (and eventually became eliminated) became replicated later than bulk DNA, but earlier than MDSs.

### The transient expression of histone variant H3.4 and H3K27me3 overlaps with the occurrence of polytene chromosomes in macronuclear anlagen

Interestingly, three histone variants of interest—H3.7, H3.5 and H3.4—exhibited differential developmental expression patterns (Fig. 4c), which at least for H3.4 fitted well with the hypothesized differential availability during heterochromatin formation. In contrast, H3.7 is an hyperacetylated replacement variant being only transiently incorporated very early in developing macronuclei [12]. Its function might be related to the replacement of micronucleus-specific H3.8 and thus to the loosening of the highly compacted zygote nucleus chromatin formed after haploid micronuclei fusion. Variant H3.5, though becoming gradually enriched in developing macronuclei, occurs in macronuclei throughout the life cycle [12]. Importantly, although both variants, H3.4 and H3.5, possess a conserved lysine 27 and were expressed in almost equal amounts 30 h PC and thereafter during further development, our data suggested that H3.4 is the preferential substrate for histone KTM activity directed to lysine 27. In particular, two H3 variants in *Stylonychia*—H3.4 and H3.8—harboured an identical motif (H3.4: K_27_TAPA; H3.8: K_32_TAPA) reminiscent of canonical H3 that was recognized by anti-H3K27me3 antibodies, if lysine 27 in H3.4 or lysine 32 in H3.8 were trimethylated (compare Additional file 1: Figure S3B and [12, 14]).

H3.4 is differentially expressed during chromosome polytenization. Although the K27me3 epitope is also present in H3.8, this 20 kDa H3 variant is restricted to micronuclei [12]. Other H3 variants, which were investigated in macronuclear anlagen, do not exhibit H3K27me3 (1. H3.7, a development-specific 20 kDa variant has no conserved motif homologous to H3K27; 2. H3.5 and H3.5 are abundant in developing and mature macronuclei at stages when H3K27me3 is not present.). Both variants H3.5 and H3.3 have deviant epitopes adjacent to K27 with additional amino acid substitutions/insertions (H3.5: K_27_TAQVAQ/H3.3: H3.5: K_27_STNVN), which most probably influence its availability as a KTM substrate or readout by putative effector proteins. The expression of other H3 variants seemed to be insignificant with respect to macronuclear development.

We observed that the expression of H3.4, which supposedly was target for K27 trimethylation, was marginal in vegetative *Stylonychia* and until 20 h PC. Then its expression increased massively during early polytenization from 20 to 30 h PC, before dropping again after 50 h PC (Fig. 4c). Thereby, the availability of both H3.4 seemed to outlast the process of heterochromatin formation, whereas other genes putatively relevant for condensed chromatin structure formation became only transiently expressed within a restricted critical time frame (~ 20 to 40 h PC), i.e. the putative histone KMT SET4 and the histone chaperones ASF1, CAF1 and HIR1, furthermore two histone H1 variants (Additional file 1: Figure S4). They all decreased from 40 h PC. We assume that a temporally restricted activity of either the H3K27me3 activity or the chromatin assembly machinery in form of histone chaperones could lead to the preferential enrichment of H3K27me3 nucleosomes at bulk DNA sequences, while MDS replication was stalled through PIWII1/27nt-RNA. Later, after replication at MDSs would have become completed and H3.4 or H3.5 became assembled into these sequences, a lack of H3.4K27me3 activity could have counteracted the formation of MDS-associated heterochromatin. Alternatively, but not mutually exclusive, a lack of histone chaperone activity could suppress the assembly of new nucleosomes into MDS chromatin. While this remains unknown, these proposed mechanisms would depend largely on replication timing and the coordinated availability of involved factors. We thus interrogated whether PIWI1/27nt-RNA complexes could have indeed the mechanistic potency to interfere with DNA replication and thus to be involved in the control of replication timing.

### Small RNAs and Argonaute/oligonucleotide complexes impair DNA replication in vitro

To elucidate the influence of 27nt-RNAs and PIWI1 on the activity of several polymerases, we tested in vitro:

1. Whether 27nt-RNAs can modify DNA replication using *Pfu* and *Taq* polymerases for PCR (Fig. 5a);

**Fig. 5:**
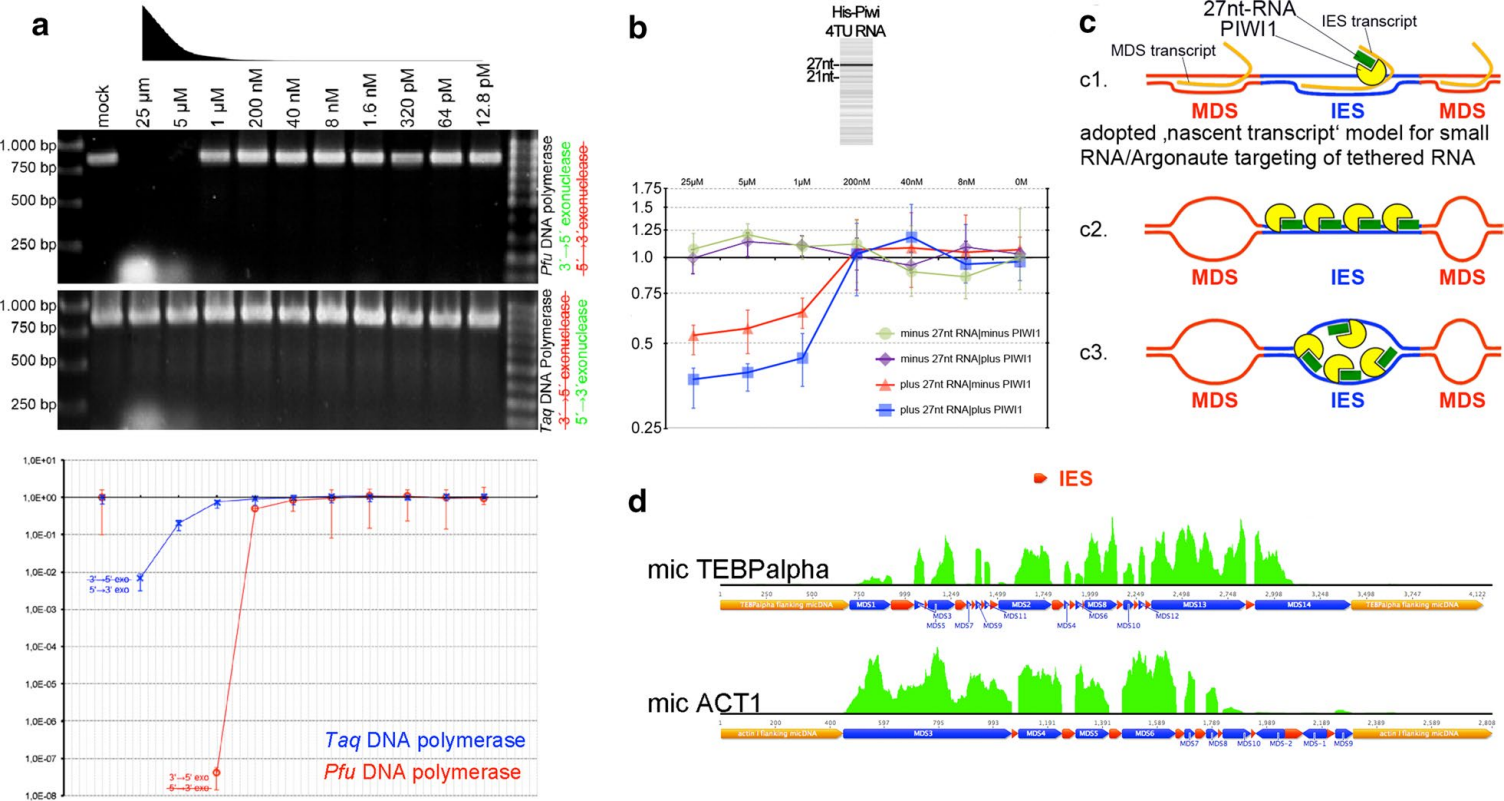
Results of ‘RNA-induced DNA replication interference’ assays. **a** Effects of 27nt-RNAs using *Pfu* or *Taq* polymerases after end-point-PCR and agarose gel electrophoresis (top) or after qPCR (bottom). **b** Effects of 27nt-RNAs alone or in combination with PIWI1 on linear DNA amplification in a *Klenow* reaction were assayed via qPCR. **c** Hypothetical models on sequence-specific targeting through Argonaute/PIWI-RNA complexes (blue: IES, red: MDS, yellow: PIWI1, green: 27nt-RNA, orange: tethered transcript [**c1** only]): **c1** According to the ‚nascent transcript model, 27nt-RNA/PIWI1 complexes could target tethered IES-originating transcripts, which would be reminiscent of observations made in divergent eukaryotes, such as *S. pombe*, *C. elegans* and *A. thaliana* (reviewed in [38]). Alternatively, we assumed that 27nt-RNA/PIWI1 complexes could interact with dsDNA (**c2**) or via base-pairing with ssDNA, possibly when it occurs in a replication bubble (**c3**). **d** Mapping of mRNA reads purified 20 h PC on micronuclear model genes reveals that IES (red bars) are sharply omitted
2. Whether oligonucleotides (5′-phosphorylated 21nt-DNA or analogous 21nt-ssRNA) and a heat stable Argonaute protein—*Thermus thermophilus* Argonaute (TtAgo)—can modify DNA replication in a qPCR (Additional file 1: Figure S5);
3. Whether 27nt-RNAs in combination with *Stylonychia* PIWI1 can modify linear DNA amplification in a *Klenow* reaction (Fig. 5b).

As template for all assays we used a cloned 856 bp PCR fragment from the PCNA1 gene on the nanochromosome Contig13765—a differentially expressed PCNA-form during macronuclear development. To minimize the possibility that PCR primers were bound by Argonautes, we used excess length oligos with 5′-non-matching nucleotides forming hairpin loops (Additional file 1: Figure S6A). We used *Pfu* or *Taq* polymeases in different reactions to consider the effects of their specific exonuclease activities (*Pfu*: 3′ → 5′; *Taq*: 5′ → 3′). When mixes of serially diluted 27nt-RNAs (25–12.8 pM) matching PCNA1 were added to the PCR reactions (Additional file 1: Figure S6A), we observed impaired amplification at higher 27nt-RNA concentrations using *Pfu* polymerase and end-point PCR, whereas a specific band appeared at 1 µM or below (Fig. 5a; top). Nonsense 27nt-RNAs had no effect on the PCR reaction (Additional file 1: Figure S6B). Using *Taq* polymerase under identical conditions, a band became visible in all reactions showing that 5′ → 3′ exonuclease activity counteracted replication impairment through 27nt-RNAs. Nonetheless, qPCR analyses under similar conditions demonstrated that 27nt-RNAs from 25 µM to 1 µM in fact impaired DNA amplification, even if *Taq* polymerase was used (Fig. 5a; bottom). Comparatively, the efficiency of PCR impairment measured by qPCR was far stronger, when *Pfu* polymerase was used at 25–40 nM. These experiments showed clearly that 27nt-RNAs in principle could impair DNA amplification using different polymerases in vitro. Remarkably, at least *Taq* polymerase can exhibit strong strand displacement activity, i.e. it displaces downstream encountered DNA. Although we observed a clear dependency of DNA replication interference on the 27nt-RNA concentration used, it does not seem that displacement activity can also lead to the degradation of RNA in RNA/DNA hybrids.

To discover whether PIWI1 and 27nt-RNAs can block DNA replication, we synthesized PIWI1 with ‘universalized’ genetic code (since ciliates use an alternative genetic code) and N-terminal His-tag. Subsequently, we cloned His-PIWI1 into pCMV-TNT (Promega) for in vitro expression. Prior to assaying the inhibitory potency on replication, we tested whether in vitro produced His-PIWI1 can bind 27nt-RNAs. We therefore incubated His-PIWI1 with 4TU-RNA mixture obtained after purification of RNA from 4TU-labelled exconjugants, followed by UV cross-linking (365 nm) and PIWI1-RNPs pull-down with PIWI1-specific antibodies [14]. Using the Small RNA Analysis Kit (Agilent) for microcapillary electrophoresis of RNA a faint 27nt band purified from PIWI1 immunocomplexes could be detected (Fig. 5b, top). To assay the efficiency of DNA replication in the presence of PIWI1 and 27nt-RNAs, we set up various *Klenow* reactions using pGEM-T-easy-PCNA1 as template. Here, we used the T7 primer-binding site in pGEM-T-easy for priming of linear DNA amplification and a modified T7 primer with non-matching, hairpin-forming additional 5′-nucleotides. Differences in the amount of amplified PCNA1 were then detected using qPCR and PCNA1 primers (Fig. 5b, bottom). The addition of PIWI1 and 27nt-RNAs in a 1:2 molar ratio revealed a significant reduction of amplified DNA between 25 and 1 µM, and this effect was apparently stronger as the effect of 27nt-RNAs alone. The addition of PIWI1 alone, in contrast, did not differ from controls without PIWI1 and 27nt-RNA.

Such ‘replication interference’ through oligonucleotides shown in vitro would provide a mechanism, which could underlie the control of replication timing during polytenization in vivo, and this could be a prerequisite for the temporally coordinated deposition of histone variant-containing nucleosomes within a given timeframe. Notably, according to the current doctrine on the RNAi-induced transcriptional silencing (RITS) for several model eukaryotes (e.g. *S. pombe*, *C. elegans*, *A. thaliana*), it is not assumed that Argonaute/sncRNA complexes bind directly to a DNA target. Rather, they apparently bind via base-pairing to tethered nascent transcripts, which origin from the target regions. Binding of such transcripts through Argonaute/scnRNAs leads to the localized recruitment of chromatin modifiers [38]. For a hypothetical ‘nascent transcript’ IES-targeting model in *Stylonychia* (Fig. 5c1), we assumed that a putative nascent transcript from a micronuclear MDS locus should contain sequences complementary to IESs. To test whether such a nascent transcript that origins from micronuclear genes could exist, we screened the developmental transcriptomes for reads matching to known IES sequences. Figure 5d shows that the reads mutually exclusively covered MDSs, whereas IESs were sharply omitted (here, 20 h PC). This observation did not support MDS targeting via a nascent transcript, thus pinpointing to a possible 27nt-RNA/dsDNA interaction (Fig. 5c2) or direct 27nt-RNA/ssDNA base-pairing in an open replication bubble (Fig. 5c3), with the latter being favoured through our finding that 27nt-RNAs became enriched, when RNA/DNA hybrids were pulled down with specific antibodies.

### 27nt-RNAs can impair DNA replication in the replication band of vegetative *Stylonychia* in vivo

**Fig. 6:**
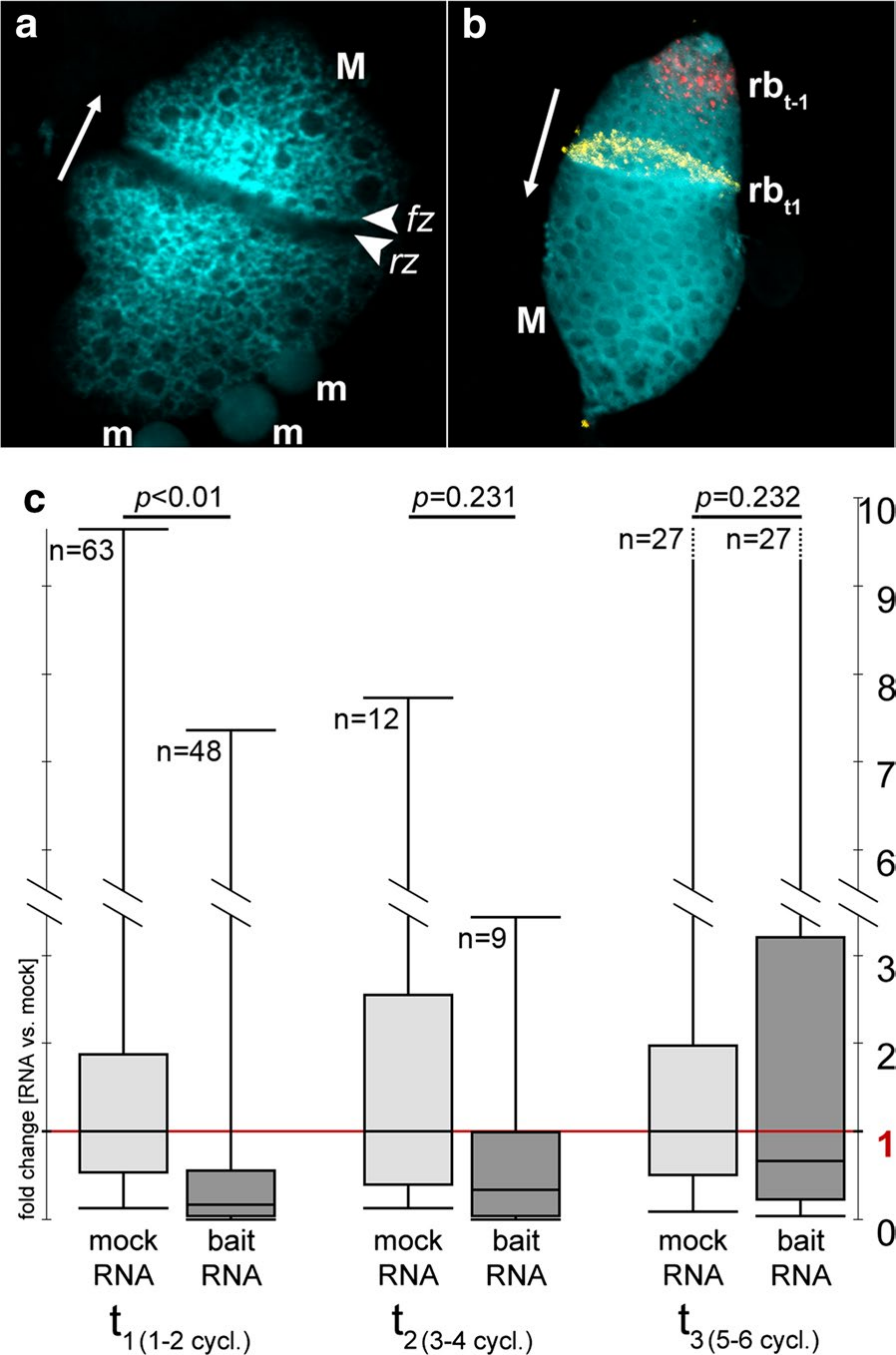
The nanochromosome copy number in vegetative macronuclei is an indicator for the existence of ‘RNA-induced DNA replication interference’ following S-phase treatments. **a** Replication in vegetative macronuclei (M) takes place in replication bands comprising of a forward zone (fz) with high DNA concentration and a rear zone (rz) with low DNA concentrations. The fz determines the direction of migration (arrow). Replication bands do never appear in micronuclei (m). **b** The motility of chromatin in macronuclei is strictly constrained, as revealed through pulse-chase-pulse experiments, where newly replicated DNA was 30 min pulse-labelled with 5-iodo-2′-deoxyuridine (red signals), followed by a 2 h chase and then a 30 min pulse with 5-chloro-2′-deoxyuridine (yellow signals): The signals from the first pulse remain within a restricted area even after prolonged observation periods/chases [20]. The arrow indicates the migration direction. **a**, **b** Top-Pro-3 was used for DNA counterstaining, false colours were used for illustration. **c** The chart illustrates the effects of mock/bait RNA treatment (microinjection) on the copy numbers of targeted nanochromosomes at 3 different time periods (*t*_1–3_). The chart combines the data from all (target) experiments. The mock median was used as reference (onefold change) for the calculations of relative fold changes. Data was obtained from 48 independent qPCR reactions in triplicate (mock RNA) or 64 independent qPCR reactions in triplicate (bait RNA), whereby three different reporter amplicons were evenly distributed for bait RNA PCR reactions

To evaluate whether the ‘RNA-induced DNA replication interference’ could in principle exist in vivo, we exploited another feature characteristic for spirotrichous ciliates—the linear spatiotemporal progression of replication during the S-phase of vegetative macronuclei within a migrating morphological structure called ‘replication band’ (Fig. 6a). Replication bands in *Stylonychia* macronuclei are disc-shaped accumulations of hundreds of synchronously firing replication foci, which appear at the distal tips of the macronuclei at the onset of S-phase and migrate thereafter to the centre of the macronucleus. Hereby, the motility of macronuclear chromatin is strictly constrained, ensuring that each nanochromosome becomes evenly replicated and its copy number remains preserved (Fig. 6b) [20]. We described above that 27nt-RNAs were absent in vegetative *Stylonychia*, whereas a low level 21–22nt-RNAs seemed to exist over the entire life cycle (Fig. 2). Concomitantly, at least one PIWI-domain protein is constitutively expressed (PIWI2, see Fig. 1 and Additional file 1: Figure S7). A prediction of the ‘RNA-induced DNA replication interference’ concept would imply that the copy numbers of specific nanochromosomes became influenced, if 27nt-RNAs targeted them during replication. Therefore, we microinjected several 27nt-RNAs (Additional file 1: Table S1) specifically targeting genes (bait RNA), which become differentially expressed during macronuclear development (i.e. H3.7 [Contig610.g675]; MDP2 [Contig9714.g10391]; PCNA1 [Contig13765.g14680]). For control a mock oligo was injected not targeting any known *Stylonychia* macronuclear DNA sequence. We assumed that during vegetative growth a change in the copy numbers of these genes, including PCNA1—one out of three PCNA genes in *Stylonychia*, which is only expressed during macronuclear development, would not affect any cellular function during vegetative growth. Importantly, in non-synchronized vegetative *Stylonychia* cells the copy numbers of several nanochromosomes were studied extensively previously, and these experiments did not give any hint for a theoretically imaginable DNA degradation event under RNAi influence [17].

Then, using qPCR on single cells we compared the relative copy numbers of the targeted nanochromosomes with copy numbers in control cells, wherein nonsense-27nt-RNAs (mock) were injected, at three successive time points. No conspicuous differences or restrictions in growth between mock and bait RNA-treated cells were observed: Following microinjection, cells were grown individually in microtiter plates and inspected in daily routine. The observed growth rates were 2.31-fold/d for bait RNA-treated cells and 2.18-fold/d for mock RNA-treated cells. Intriguingly, we observed a significant reduction in the copy numbers of targeted nanochromosomes between the 1st and 2nd replication cycles after microinjection (mean fold change: 0.17314, *p *< 0.01) (Fig. 6c). When we assessed the copy numbers of targeted nanochromosomes in the course of continuous cycles (3rd to 4th/5th to 6th), we observed an attenuation of the copy number effects observed after the first two cycles, whereas a weak interference effect on the targeted nanochromosome copy numbers still appeared to be recognizable. Taken together, this observation substantiated the initial prediction that 27nt-RNAs targeting specific nanochromosomes during replication would influence their copy numbers. Consequently, these experiments provided first evidence that an ‘RNA-induced DNA replication interference’-mechanism is possible in principle in vivo and could therefore exist.

## Discussion

### Precise timing is essential for two non-coding RNA species that act cooperatively in IES excision, MDS reordering and the protection of premature macronuclear genes

Our data provide a line of evidence that PIWI1-bound 27nt-RNAs target MDSs via RNA/DNA base-pairing during polytenization, which encompasses a series of 5–6 DNA replication cycles in *Stylonychia* [1]. Moreover, binding of small RNAs to DNA could physically influence the efficacy of DNA replication, which does not take place synchronously during polytenization of different germline sequence classes. These observations are the key findings of this work and drive the proposed ‘RNA-induced DNA replication interference’ hypothesis.

Thereafter, RNA-induced DNA replication interference would require a series of synchronized events, i.e. briefly:

1. The termination of MDS module reordering in scrambled micronuclear genes and the removal of interrupting IESs.
2. Targeting/protection of MDSs by PIWI1-bound 27nt-RNA complexes, which leads to locally stalled DNA replication during chromosome polytenization. This sequence protection might lead to the omission/attenuation of H3K27me3 enrichment at MDSs.
3. Subsequent enrichment of H3K27me3 at bulk DNA sequences leading to a unique chromatin structure, which is required for DNA elimination.

With respect to scrambled gene reordering and IES removal, there is growing consent that a ‘template’ non-coding RNA species that acts on micronuclear genes might occur within a narrow timeframe prior to bulk DNA elimination. It is unknown whether this timeframe overlaps with early chromosome polytenization. The ‘template’ RNA is not yet fully characterized, but it is presumably involved in MDS reordering, IES excision, nanochromosome copy number determination and telomere addition to immature nanochromosomes [16–19]. It is difficult to imagine that micronuclear genes in a scrambled disorder already start unimpeded serial polytene chromosome replication unless IES excision and reordering are completed, although intermediates of MDS reordering and IES excision were previously identified at the onset of polytenization. However, the temporal uncertainty of these analyses comprised 4–5 h [10]. It is thus well possible that IES excision takes place separately and immediately before polytenization starts in a time window that could not yet be resolved exactly. Notably, it cannot be absolutely ruled out that, at least partly, unscrambling and MDS protection through PIWI1/27nt-RNA complexes could be spatiotemporally and functionally intermingled processed, which synergistically block the enrichment of H3K27me3 on MDSs. Our observation that IES excision on micronuclear model genes could take place even though PIWI1 was silenced in RNAi experiment speaks against this objection, however.

Whereas it is currently technically hardly possible to achieve a better resolution of the exact timing, we assume that 27nt-RNAs act subsequently in the course of serial polytenization on the micronuclear gene loci, which harbour MDSs, thereby protecting them from being entangled in subsequent elimination of bulk DNA. The most plausible scenario is that 27nt-RNAs protect genes as soon as IES excision and MDS reordering have been completed, safeguarding that these immature macronuclear genes remain protected from heterochromatin formation. In theory, RNA/DNA base-pairing of 27nt-RNAs within reordered MDSs would require single-stranded DNA which would be given in a replication bubble, i.e. during polytenization (Fig. 5c3). We observed that during this critical time frame PIWI1 in fact swamps the developing macronucleus probably loaded with 27nt-RNAs, which origin from the parental macronucleus. PIWI1 obviously takes part in the transnuclear trafficking of 27nt-RNAs [14], and it is thus the physical transmitter of parental genome partitioning information.

Whereas the knock-down of PIWI1 by RNAi does not touch the excision of IESs (Fig. 3a), the elimination of bulk DNA became substantially impaired (Fig. 3b). This observation is coherent with respect to the chronology of sequence reduction, where the appearance of PIWI1 at MDSs in developing macronuclei lags behind the removal of IESs. Scattered earlier observations list several other features, which are affected in PIWI1-minus *Stylonychia*. These include the persistence of the parental (old) macronuclei, which could indicate that the translocation of 27nt-RNAs therefrom is blocked, and the developmental arrest of macronuclear anlagen [39]. Furthermore, observed effects in PIWI1-minus *Stylonychia* within developing macronuclei comprise the observation that the micronucleus-specific 20 kDa histone variant H3.8 (formerly ‘protein X’) that usually becomes replaced after synkaryon/zygote formation persists in developing macronuclei, whereas 15 kDa H3 is detectable in Western blots, but does not become methylated at lysine 27 [13]. We observed further that the expression of the histone variants H3.3 and H3.5, which occur in developing and mature macronuclei, is impaired on the mRNA level [12]. It is very well in agreement with the proposed chronology that most of these effects take place downstream of IES removal. This might explain why bulk DNA removal does not take place, even though PIWI1/27nt-RNA complexes do not directly interact with those sequences: It seems convincing that the complex molecular turnover during polytene chromosome replication, which directly and centrally involves PIWI1, could require a S-phase termination checkpoint to safeguard that replication was terminated properly. If this checkpoint cannot be passed in PIWI-minus cells, downstream effects do not take place, i.e. bulk sequence association with H3K27me3 and eventually programmed DNA diminution.

Eventually, it is imperative that all MDSs must be marked for preservation and bulk DNA must be assembled into a condensed chromatin structure at the time point, when DNA replication/chromosome polytenization is terminated. This expectation is obviously fulfilled: Microscopy of polytene chromosomes demonstrates that PIWI1 and H3K27me3 exhibit mutually exclusive staining patterns. In particular, PIWI1 occurred in non-condensed polytene chromosome regions, whereas H3K27me3 was associated with condensed chromatin bands. We assume that this pattern could be achieved, if MDSs replication became locally and transiently stalled (protected), whereas the continuous replication of unprotected bulk DNA regions would be coupled to the incorporation of specific, temporally available histones, which shape a discrete chromatin structure that determines associated sequences for elimination. Due to technical restrictions, there is currently no close reasoning for all parts of the proposed concept. But in agreement with the requirements of our proposed concept we showed that during polytenization not all sequences become synchronously replicated. In particular, sequences being slated for elimination become earlier polytenized than MDSs during macronuclear development, e.g. the maximum of copy number of the bulk DNA model sequences MaA81 and Stad5 is reached approx. 10–20 h earlier than for the micronuclear gene models TEBPalpha and ACT1 (Fig. 3b). This suggests that bulk DNA replication surges ahead of micronuclear gene replication. We propose that during the bulk DNA replication events specific, temporally available histone variants could be assembled in a replication-dependent manner. Interestingly, we provided evidence that the post-translational modification (H3K27me3) of an involved H3 variant occurred in the cytoplasm prior to its assembly into polytene chromosomes. Again, technical restrictions do not allow closer reasoning with respect to the precise timing of specific nucleosome assembly within the critical timeframe, which could be influenced by the limited availability of H3K27me3, a histone chaperone, an effector that binds H3K27me3 or other chromatin assembly factors or signals. However, we provide robust evidence that there is an expression peak of the histone variant H3.4, which is probably the only H3 variant that accumulates de novo in developing macronuclei and is a substrate for K27me3. In contrast, H3.5 and to a much lower extent H3.3 are already abundant in very early developing macronuclei and in mature macronuclei. Both early macronuclei anlagen and mature macronuclei are completely devoid of H3K27me3 [12, 14].

Importantly, whether it is H3.4 or not, a histone H3 post-translationally modified by K27me3 in the cytoplasm becomes most probably de novo incorporated into polytene chromosomes. Indisputably, the observable result is the formation of an alternating, mutually exclusive pattern of H3K27me3, which is associated with condensed chromatin bands but separated from PIWI1-associated, uncondensed chromatin. This result seems to be fully compatible with the scenario that under the condition of transient MDS sequence protection a limited dose of H3(.4)K27me3 could be available (or other chromatin assembly factors that influence the deposition of H3K27me3). Other conceivable scenarios are provided as Supplementary data (Additional file 1: Table S2). These seem to be less probable to hold, since the observed chromatin signatures on polytene chromosome fall short of the expectations concluded from those alternative scenarios (see Additional file 1: Table S2 for an overview).

However, since at least H3.5, H3.4 and also H3.3 are readily available during selective bulk DNA polytenization, it seems improbable that purely H3.4K27me3 becomes associated with those sequences. Moreover, for MDSs we noted above that the sequences containing the precursors of the non-transcribed subtelomeric regions of the nanochromosomes were omitted from sncRNAs. This indicates that not the entire body of MDSs in the developing macronucleus became targeted. Plain-talking, MDS protection might not depend on the full coverage of their entire sequences. Local PIWI1/27nt-RNA binding might also nucleate the protection of adjacent sequences within a micronuclear gene locus. On the other hand, adopting a differential chromatin structure might not depend on a ‘pure’ histone variant/PTM signature for bulk DNA sequences. We speculate that variances in both mechanisms could matter for the efficacy of both processes, i.e. sequence protection and sequence reduction. Therefore, we note that a differential H3K27me3 signal strength could be achieved in theory: When compared with MDSs, each replication cycle wherewith bulk DNA replication surges ahead of MDS replication should result in a doubling of H3.4K27me3 association with bulk DNA (or exponential increase with each additional cycle in advance). While being speculative, these scenarios leave open whether differential efficacy of MDS protection, possibly through differences in 27nt-RNA coverage (which correlate with mRNA levels), could lead to differences in mature MDS preservation. This could contribute to a rough predetermination of nanochromosome copy numbers. In this case MDS protection could be scalable through the mRNA copy number in the parental macronucleus. Notably, the possibility that the parental mRNAs could be the substrates for the synthesis of 27nt-RNAs is supported by the observed tight correlation of 27nt-RNA/mRNA copy numbers and by the observation that 27nt-RNAs cover the transcribed region of nanochromosomes only.

Apart from these speculations we summarize that IES excision and MDS reordering, which do not depend on PIWI1, must take place within a restricted time window prior to bulk DNA elimination. This time window possibly opens shortly before the onset of polytene chromosome formation. Subsequently, MDSs become protected and this depends on PIWI1. MDS protection must be achieved during polytene chromosome replication before bulk DNA adopts a heterochromatin structure through H3K27me3 and sequence elimination can start. Thus, the onset of DNA diminution possibly requires an S-phase termination checkpoint. We conclude that a series of precisely timed events is crucial for the tight coordination of these processes and that ‘RNA-induced DNA replication interference’ can in theory provide a mechanistic explanation. Below, in our further discussion we consider additional results to provide stronger support to the plausibility of the RIRI hypothesis.

### The concept of ‘RNA-induced DNA replication interference (RIRI)’ through protective 27nt-RNAs provides a mechanism to control a timeframe, wherein the histone variant H3.4 and H3K27me3 become selectively associated with bulk DNA, and a line of evidence support its existence in vivo

We showed in vitro that 27nt-RNAs have the potency to block the DNA replication activity of several DNA polymerases in the vicinity of base-paired homologous sequences. Our assays using 27nt-RNAs alone, 21nt-phDNA or both oligonucleotides in combination with Argonaute homologs (TtAgo [see Additional file 1: Figure S5] and PIWI1) show that DNA replication interference leads to impaired DNA replication in vitro. Although we cannot exclude that PIWI1 in *Stylonychia* is mainly connected with 27nt-RNA trafficking between parental macronuclei and anlagen, our data suggest this Argonaute homolog could strengthen the DNA replication interference effect during chromosome polytenization in developing macronuclei as it appeared to be the case at least in a *Klenow* reaction assay. This was reminiscent of reports on non-slicing Argonaute/guide RNA complexes, which function as ‘road blocks’ to inhibit the translation of mRNAs in absence of other auxiliary proteins: In almost all groups of organisms, RNase H degrades selectively the RNA strand in RNA/DNA hybrids. The PIWI-domain in slicing Argonaute proteins in eukaryotes and archaea includes an RNase H-like active site capable for guide strand binding and slicing of RNA/RNA hybrids [40–42]. Frequently, Argonautes loaded with guide RNA function without the contribution of other proteins, whereas—apart from slicing activity—sterical blocking (‘road block’) was reported for catalytically inactive Argonautes, e.g. in AGO1/microRNA (guide)/mRNA (passenger) complexes on the ribosome [42]. Remarkably, a unidirectional sterical DNA barrier function by Tus-Ter affects replication fork progression in *E. coli*. Interestingly, it was shown that the Tus-Ter only transiently blocks the replisome in the non-permissive direction [43], which is reminiscent of our hypothesis for *Stylonychia*. We thus believe that a transient DNA replication ‘road block’ is the most plausible explanation for 27nt-RNA/PIWI1 complexes.

Notably, with its conserved DDH triad (D555, D626, H758) and a putative glutamate finger (including E590 or E594) the PIWI/RNase H-like domain *Stylonychia* PIWI1 can most probably adopt a conformation that is reminiscent of slicing Argonautes, although not all Argonautes having the complete catalytic tetrad are competent for target cleavage [42]. However, DNA slicing would be incompatible with the concept of MDS protection. Our success to purify 27nt-RNA/DNA hybrids suggests that these complexes are stable and thus functionally relevant. We assume that the putatively conserved slicing activity of PIWI1 points to a hypothetical slicing function directed to RNA/RNA duplexes, which could occur in the course of 27nt-RNA biogenesis.

While the above-described DNA interference experiments were performed using naked DNA, we were also able to utilize vegetatively proliferating *Stylonychia*, in particular the replication of nanochromosomes in the macronucleus, as a model system, where DNA sequences become replicated in the natural context of chromatin and nuclear architecture in vivo. We observed that microinjection of 27nt-RNAs was sufficient to impair the proper replication of targeted nanochromosomes, which transiently led to their reduced copy numbers in the progeny. We believe that this experimental system provides a practicable approach to provide first evidence that RIRI could exist in vivo. However, since nanochromosome replication in vegetative macronuclei is a process different from polytenization in developing macronuclei, the results should be interpreted with some care. Notably, in contrast to PIWI1, whose expression during macronuclear development exceeds by far all other PIWI-domain proteins in *Stylonychia*, at least one other PIWI (PIWI2) seems to be constitutively expressed over the life cycle. PIWI2 is the most abundant PIWI-domain protein during vegetative growth (Additional file 1: Figure S7). However, it is unknown whether PIWI2 can bind 27nt-RNA. Thus, our experiments leave open that base-pairing of microinjected 27nt-RNA to complementary target DNA sequences alone could transiently influence/weaken DNA replication—even in the absence of PIWI. We therefore speculate that PIWI1 on polytene chromosomes could fulfil a non-slicing, stabilizing function insofar that it sterically interferes the DNA replication machinery. This scenario is reminiscent of the sterical blockage of translation through non-slicing Argonautes targeted to complementary mRNAs by microRNAs [42].

## Conclusions

**Fig. 7:**
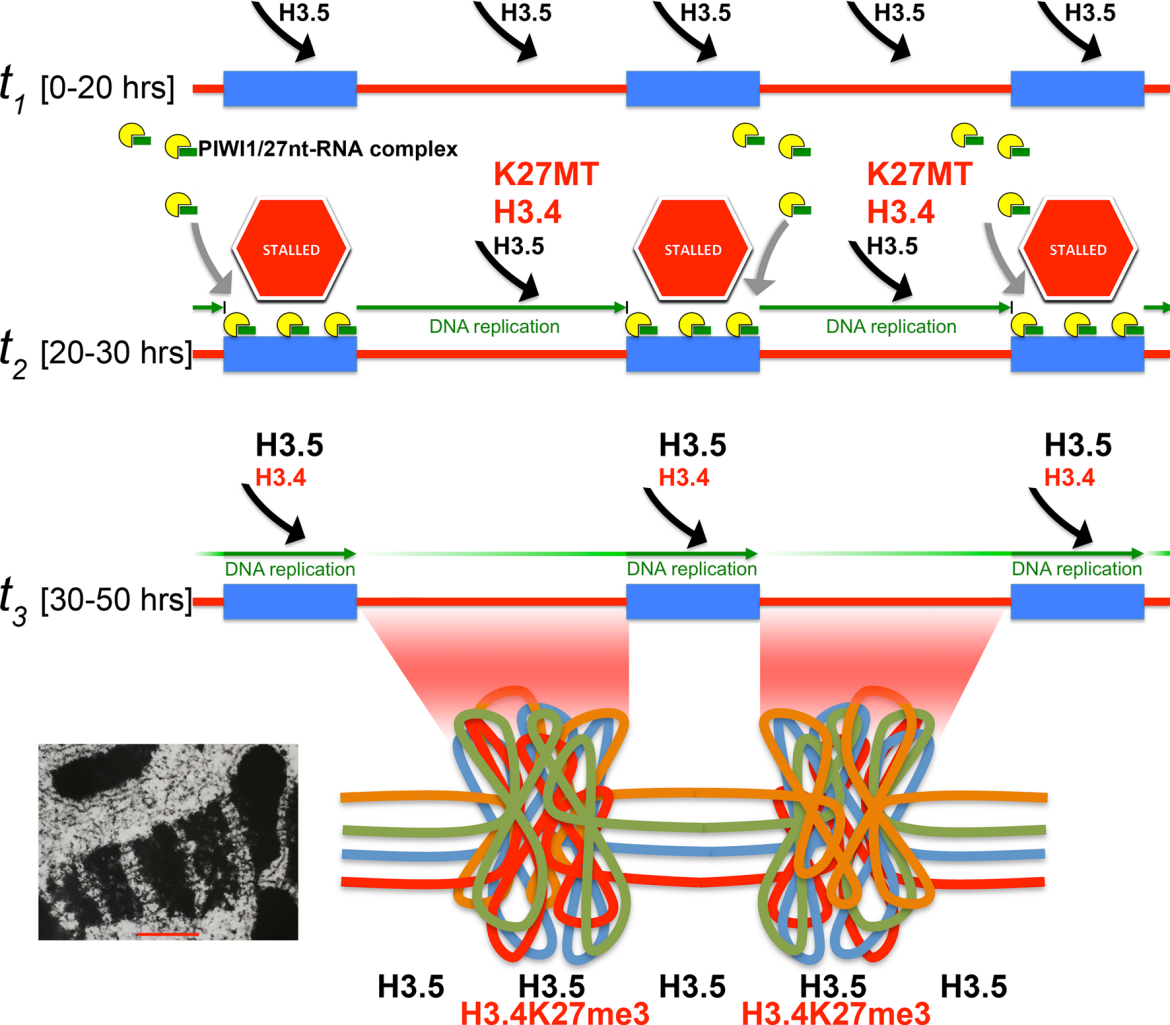
The electron micrograph (kindly donated by Ada and Don Olins) below shows a section from a polytene chromosome from developing macronuclei in *Stylonychia*. Tight heterochromatic bands are interconnected by uncondensed chromatin fibres. The right-handed cartoon is an interpretation of that micrograph within a model for ‘RNA-induced DNA replication interference’ through 27nt-RNAs. 27nt-RNAs protect DNA from being lost during macronuclear development. Accordingly, PIWI1/27nt-RNA complexes block polytenization of covered MDSs, thus limiting the replication-dependent de novo deposition of nucleosomes. Histone variant H3.4 is probably permissive for H3K27me3. H3K27me3 nucleosomes become enriched during a restricted timeframe. Eventually, this tight spatiotemporal coordination leads to the association of H3(.4)K27me3 preferentially with bulk DNA—besides H3.5 that is continuously expressed. The heterochromatin formation via H3K27me3 is limited to those regions not protected by PIWI1/27nt-RNA complexes

Taken together, the accumulated data allow us for the first time to speculate about an integrative mechanistic explanation, how 27nt-RNAs together with PIWI1 could cause ‘RNA-induced DNA replication interference’ (Fig. 7) in agreement with the hypothesis that 27nt-RNAs protect DNA from being lost during macronuclear development. Accordingly, PIWI1/27nt-RNA complexes block polytenization of covered MDSs, thus limiting the replication-dependent de novo deposition of nucleosomes. Histone variant H3.4 permissive for H3K27me3, and a specific H3K27-specific KMT activity become enriched during that timeframe. Consequently, this spatiotemporal coordination leads to the preferential association of H3.4K27me3 with bulk DNA and heterochromatin formation would be limited to those regions not protected by 27nt-RNAs. Therefore, 27nt-RNAs would act transgenerationally in the developmental establishment of defined chromatin landscapes by transferring parental genome partition information to the offspring. This mechanism would in principle be sufficient to explain how bulk intergenic DNA becomes specified for elimination and thus functionally separated from sequences that must be retained in mature macronuclei.

While current mechanistic explanations in metazoa pinpoint on specialized histone chaperones being mainly responsible for the selective chromatin deposition of histone variants [44, 45], our results highlight the possibility that also non-coding RNAs could be involved in this process in many eukaryotes. Interestingly, complex spatiotemporal DNA replication patterns in metazoa are well known [46], where actively transcribed genes tend to be replicated earlier than silent genes or intergenic DNA. These early replicated genome fractions mirror cell type-specific gene expression signatures, which are inherited to the daughter cells during mitosis. Perspectively, it will be most interesting to analyse in metazoa, whether the parental cells mRNA population might directly be involved in the establishment of gene expression patterns in the daughter cells. Mechanisms could have evolved in many metazoa sharing common ancestry with ‘RNA-induced DNA replication interference’ in *Stylonychia*, which could guide the selective deposition of replication-dependent histone variants and associated PTMs by sequence complementarity. Taken together, ‘RNA-induced DNA replication interference’ could be a not yet recognized non-coding RNA function that contributes to the regulation of replication timing.

## Additional file

**Additional file 1.** Supplementary data contains two additional tables (Tables S1/S2) and seven supplementary figures (Figures S1–S7)

## Abbreviations

4TU: 4-thiouridine
5FU: 5-fluorouridine
ASF1: anti-silencing function 1
CAF1: chromatin assembly factor 1
CldU: 5-chloro-2′-deoxyuridine
HIR1: histone regulation 1
IdU: 5-iodo-2′-deoxyuridine
IES: internal eliminated sequences
KTM: histone lysine methyltransferase
MDP: macronuclear development protein
MDS: macronucleus-destined sequences
PAR-CLIP: photoactivatable-ribonucleoside-enhanced cross-linking and immunoprecipitation
PCNA: proliferating cell nuclear antigen
PIWI: P-element induced wimpy testis
RIRI: RNA-induced DNA replication interference
(s)ncRNA: (small) non-coding RNA
TEBP: telomere-end-binding protein
TtAgo: *Thermus thermophilus* Argonaute
