# Supplementary material for: 27nt-RNAs guide histone variant deposition via ‘RNA-induced DNA replication interference’ and thus transmit parental genome partitioning in Stylonychia
Source: Epigenetics Chromatin. 2018 Jun 12;11:31. doi: 10.1186/s13072-018-0201-5 (PMC5996456; doi:10.1186/s13072-018-0201-5)
Supplement: Supplementary file 1 — Additional file 1. Supplementary data contains two additional tables (Tables S1/S2) and seven supplementary figures (Figures S1–S7) [file 13072_2018_201_MOESM1_ESM.docx]

**Supplementary Data:**

**27nt-RNAs guide histone variant deposition via ‘RNA-induced DNA replication-interference’ and thus transmit parental genome partitioning in *Stylonychia***

Jan Postberg^1,2,*^, Franziska Jönsson^3^, Patrick Philipp Weil^1,2^, Aneta Bulic^3^, Stefan Andreas Juranek^4^ and Hans-Joachim Lipps^3^

E-Mail addresses:

Jan Postberg: [jan.postberg@uni-wh.de](mailto:jan.postberg@uni-wh.de)

Franziska Jönsson: [franziska.joensson@uni-wh.de](mailto:franziska.joensson@uni-wh.de)

Patrick P Weil: [patrick.weil@uni-wh.de](mailto:Patrick.weil@uni-wh.de)

Aneta Bulic: [bulic.aneta@gmail.com](mailto:bulic.aneta@gmail.com)

Stefan A Juranek: [sajuranel@gmail.com](mailto:sajuranel@gmail.com)

Hans J Lipps: lipps@uni-wh.de

^1^Clinical Molecular Genetics and Epigenetics, Faculty of Health, Centre for Biomedical Education & Research (ZBAF), Witten/Herdecke University, Alfred-Herrhausen-Str. 50, 58448 Witten, Germany

^2^HELIOS University Hospital Wuppertal, Centre for Clinical & Translational Research (CCTR), Witten/Herdecke University, Heusnerstr. 40, 42283 Wuppertal, Germany

^2^Institute of Cell Biology, Centre for Biomedical Education and Research (ZBAF), Witten/Herdecke University, Germany

^4^Stefan Juranek, PhD, iPSC CRISPR Facility, European Research Institute for the Biology of Ageing (ERIBA), University Medical Center Groningen, The Netherlands

*corresponding author

**Contact information**

Dr. Jan Postberg

HELIOS Medical Centre Wuppertal

Witten/Herdecke University

Heusnerstr. 40

42283 Wuppertal

Germany

Phone: +49(0)202 8962544

Fax: +49(0)202 8962546

Mail: jan.postberg@uni-wh.de

**Supplemental Tables**

| Name | Sequence 5'→3' |
| --- | --- |
| PCNA1(856)_fw | gcgtcggcgaaccgacgtgaagcaaagctcacagaagg |
| PCNA1(856)_rv | gggacggaagcccgtcccattcatcgctgatcttgggg |
| PCNA1(103)_fw | cccaagaccgagagaaccac |
| PCNA1(103)_rv | tgatggtcacgactgaggag |
| H37(57)_fw | acyacagatgtcatgagcttcggg |
| H37(57)_rv | tgcttgagctggcatctcaa |
| MDP2(97)_fw | cattcaatgcaccacctggg |
| MDP2(97)_rv | gggcctctaccattgccatt |
| T7_fw | gcgtcggcgaaccgacgctaatacgactcactataggg |
| MaA81_1fw | gtaacccccgaacttgggaa |
| MaA81_1rv | aaagtgagcaccacgtaccc |
| MaA81_2fw | accagatggtgccccaataac |
| MaA81_2rv | ggggcatagagaatctagaatgaag |
| Stad5_fw | catcaaggaccgctattcctac |
| Stad5_rv | aaaacattcacccccaaagc |
| ACT1_flnk1_fw | aagcttcctggctaatatttttgt |
| ACT1_flnk1_rv | agcagattaaaatttttcaagtta |
| ACT1_flnk2_fw | attattatatattacgcattttaagcg |
| ACT1_flnk2_rv | ctcattgacctccttcgac |
| ACT1_MDS_fw | ggctgagtcaacaccgatca |
| ACT1_MDS_rv | ggagtcgtcaaggctggttt |
| TEBPA_flnk1_fw | tccagtccagtccgaagatg |
| TEBPA_flnk1_rv | cttagccagtcggcttcgtg |
| TEBPA_flnk2_fw | accggatgaaaaaggggtca |
| TEBPA_flnk2_rv | ccctcgatccccctatcctt |
| TEBPA_MDS_fw | ggacaaagataattcaatgctaacg |
| TEBPA_MDS_rv | tcctgagaaagcgaatgggg |
| 27nt-H3.7_RNA | ucaauucuaccacagaugucaugagcu |
| 27nt-MDP2_RNA | ugagaggacagccuagauuccaugaug |
| 27nt-mock_RNA | gaagucuagaccugcugcgggggugag |
| 27nt-PCNA_RNA1 | acuugucaauggacggauucgagcacu |
| 27nt-PCNA_RNA2 | ucgugaccaucaacucaggagaguucu |
| 27nt-PCNA_RNA3 | ugaagucgguucugggcaggucagacu |
| 21nt-PCNA_phDNA1 | ggacggattcgagcactacag |
| 21nt-PCNA_phDNA2 | tccaagatctgcaaggagctc |
| 21nt-PCNA_phDNA3 | gagttcaaaatcgataaccta |

**Table S1.** Oligonucleotides used in this study.

**Supplemental Figures**


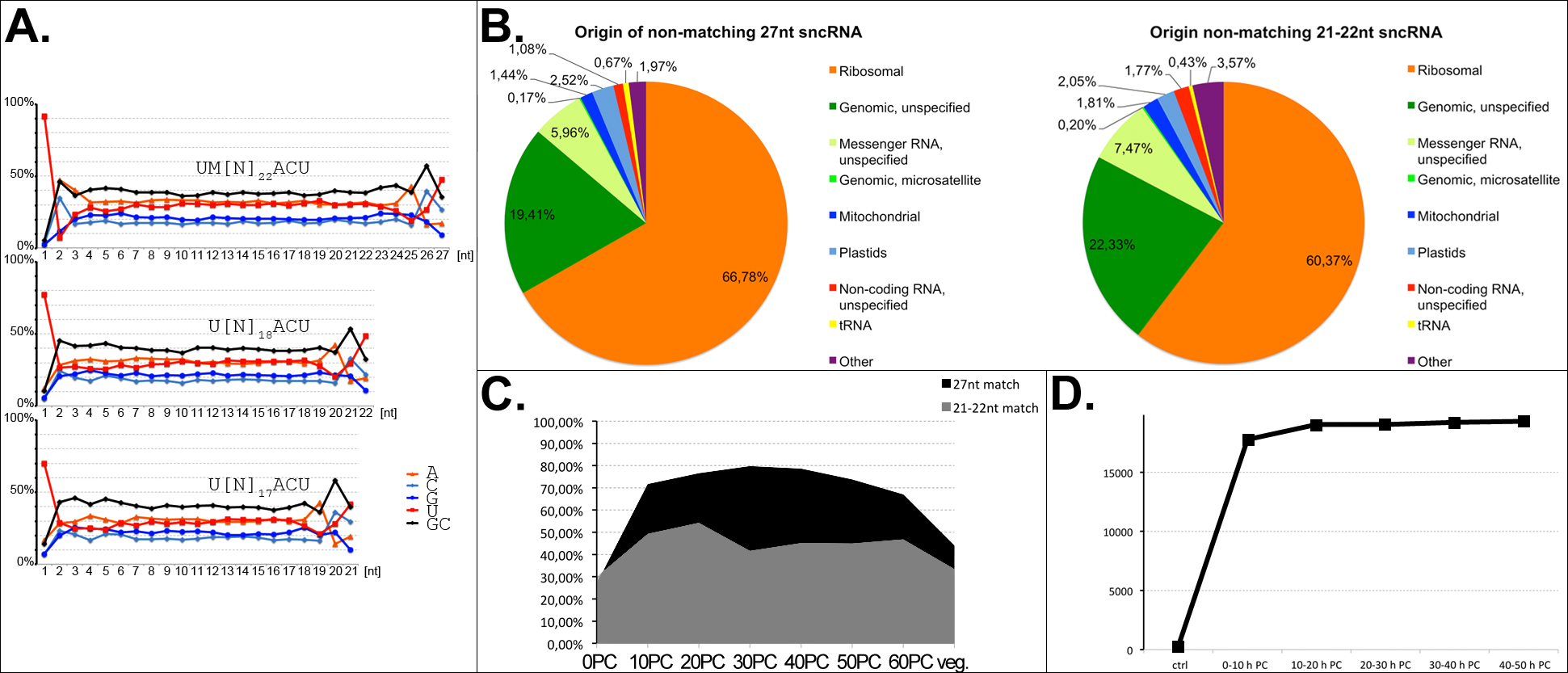


**Figure S1. Characterization of sncRNAs in *Stylonychia lemnae*. A.** 27nt-RNAs as well as 21/22nt-RNAs exhibit a conserved biased nucleotide composition at both ends. **B.** Analyses of 21-22nt-RNA as well as 27nt-RNA not matching against macronuclear contigs revealed that almost all could be assigned to probable origins, which were not micronucleus-specific sequences. **C.** The ratio of matching vs. total 27nt-RNA or 21-22nt-RNA was determined showing that a larger proportion of 27nt-RNA reads matches against macronuclear sequences during development. **D.** We counted the number of macronuclear contigs, against which 27nt-RNA matches were found at subsequent macronuclear developmental stages. At the onset of macronuclear development matches against almost all macronuclear contigs could be detected.


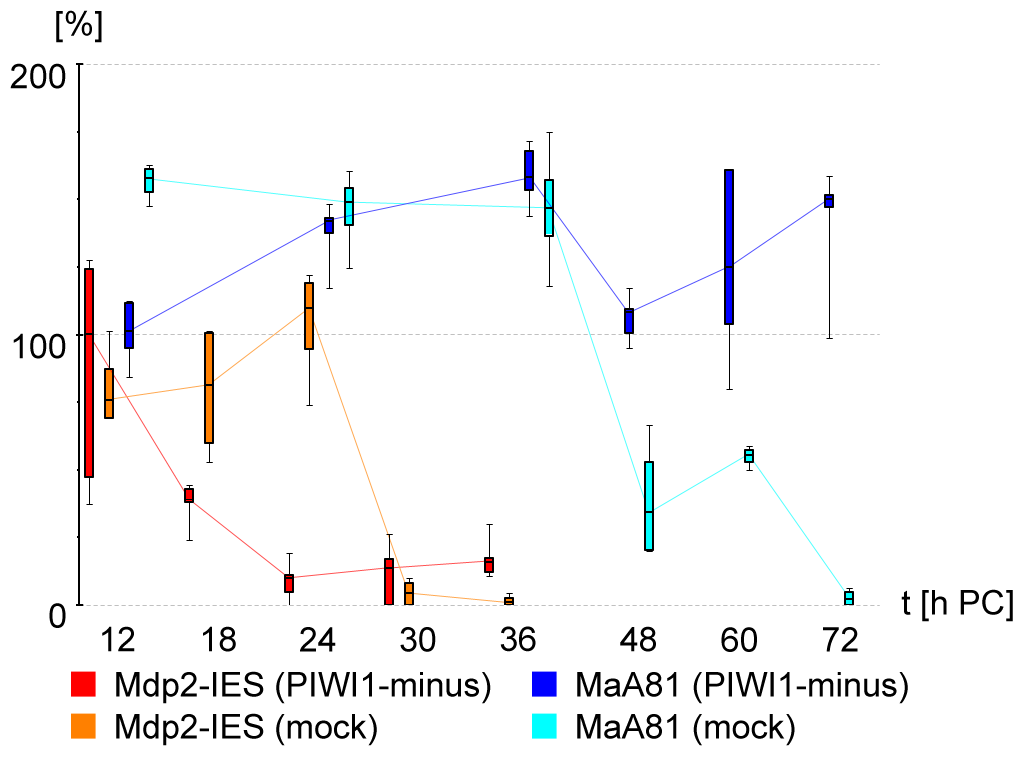


**Figure S2. Differential excision of micronuclear sequence classes analysed by qPCR.** The signal intensity was normalized using the median of the the Mdp2-IES amplicon, which was defined as 100%.


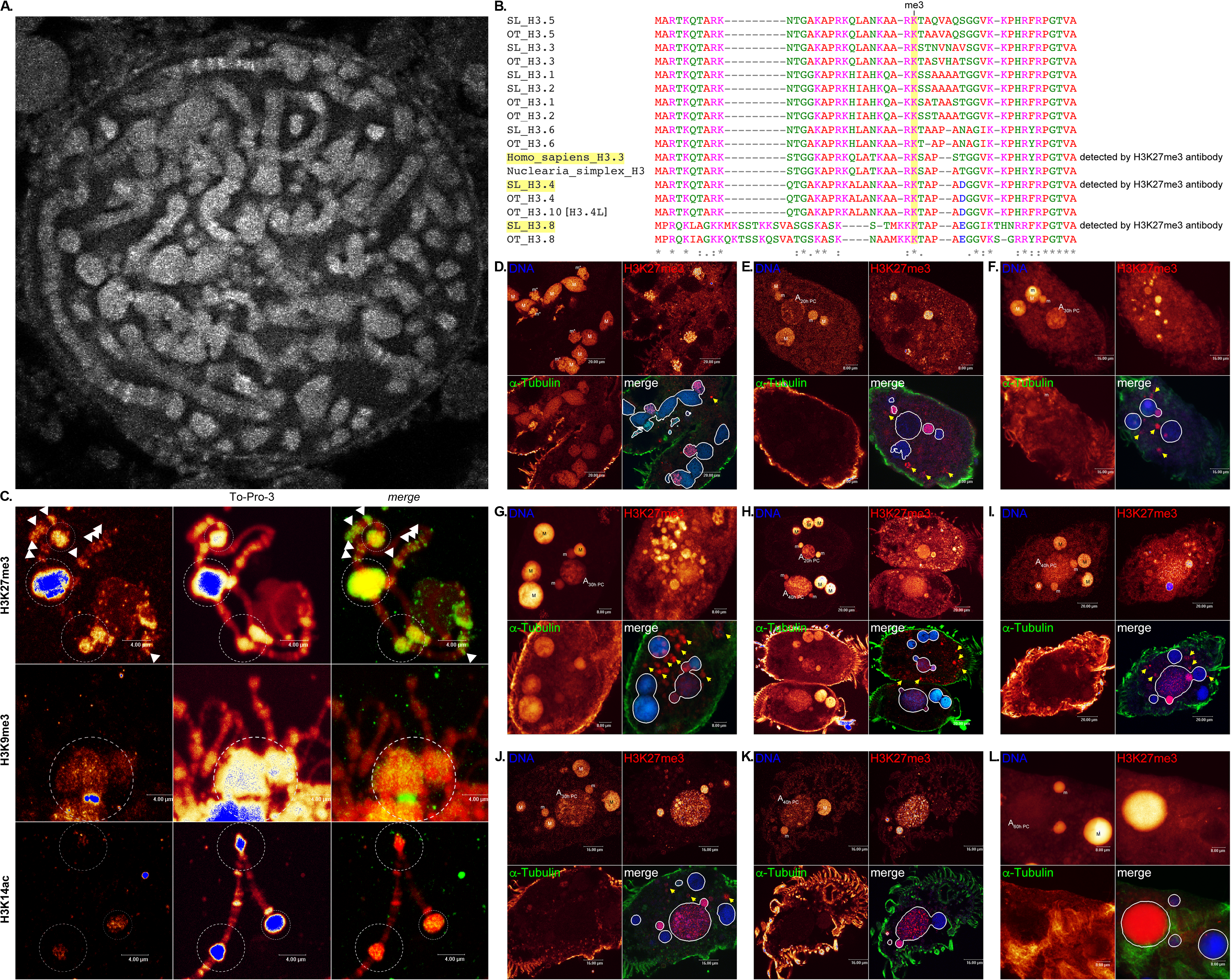


**Figure S3. Characterization of selected PTMs in polytene chromosomes in macronuclear anlagen. A.** Light optical section of a macronuclear anlage in the late stage of polytene chromosome formation. **B.** Alignment of human H3.3, *Stylonychia lemnae* (SL) and *Oxytricha trifallax* (OT) H3 N-termini demonstrating similarities and deviations in the protein sequences adjacent to H3K27. **C.** Differential patterns become visible in these projections of RAW light optical sections, when histone H3 PTMs are visualized with specific antibodies targeted to either H3K27me3 (top panel), H3K9me3 (mid panel) or H3K14ac (bottom panel). Blue colour indicates oversaturated signals. Pinched off bodies occur during DNA elimination and are marked by dashed circles. H3K27me3-positive heterochromatin bands are marked by arrowheads in the upper panel, whereas no staining is visible when H3K9me3/H3K14ac antibodies are used.

**D-L.** Cytoplasmic H3K27me3 accumulations in DNA-negative bodies (yellow arrows in the merged images; for better separation of these signals all types of nuclei were outlined with a white border) that do not co-localize with any type of nuclei suggest that this PTM becomes specifically written to H3.4 prior to its assembly into chromatin. The panel shows in (**D.**) H3.8K31me3 (H3K27me3-like) in dividing micronuclei (m*) during conjugation at the onset of sexual reproduction. A yellow arrow points in a single H3K27e3 accumulation. Cells in (**E-L.**) contain developing macronuclei (A; x hours post conjugation), micronuclei (m), fragments of the old/parental macronucleus (M) and few or multiple DNA-negative bodies that exhibit prominent H3.4K27me3 staining (yellow arrows). Most of them were observed approx. 30 hrs PC (**F-G.**), prior to the massive enrichment of H3K27me3 in macronuclear anlagen (**H-K.**). After DNA elimination H3K27me3 was still enriched in the anlagen nucleus, but H3K27me3 accumulations in DNA-negative bodies were not observed (**L.**).


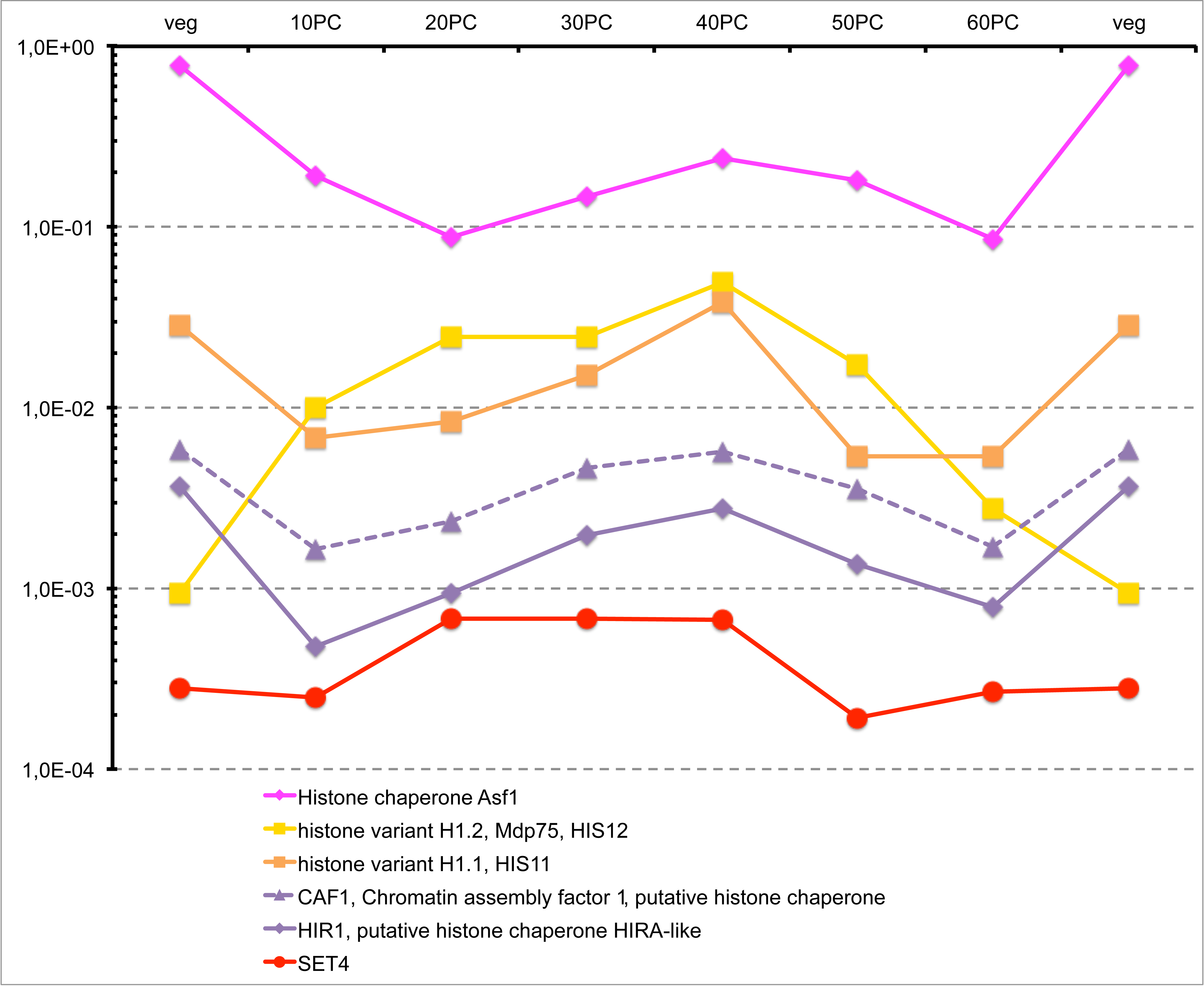


**Figure S4. Differential developmental expression of several genes of interest.** Quantification of transcripts from histone chaperone genes (ASF1, CAF1, HIR1), histone H1 variants and a putative H3K27-specific KMT (SET4) through internal normalization of each developmental time point to the histone H4 read count and normalization of each developmental time point to the read count of 15 housekeeping genes in vegetative cells. The y-axis represents the number of histone variant-of-interest per histone H4.


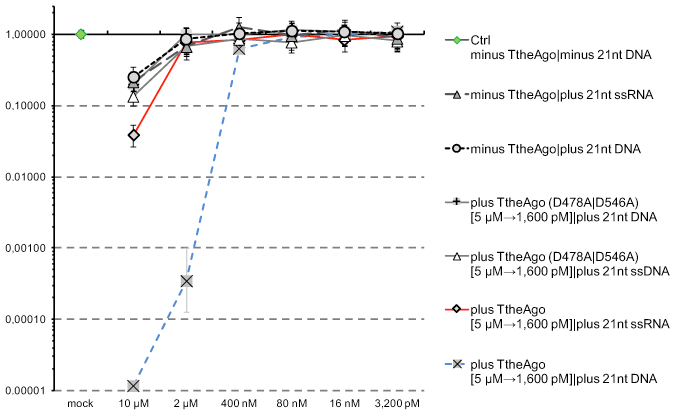


**Figure S5. Replication interference through TtAgo in combination with oligonucleotides.** Effects of 21nt-phDNAs or 21nt-ssRNAs alone or in combination with functional or non-functional TtAgo were studied, when *Taq* polymerase was used for qPCR. Importantly, PCR inhibition more efficient when compared with the use of oligos alone, when TtAgo:21nt-ssRNA was added (red line), whereas most of the inhibitory effect of TtAgo:21nt-DNA must be assigned to the target DNA cleavage activity of this complex (blue dashed line).

In particular, we tested whether heat stable TtAgo can stabilize or strengthen the replication-interference effect of noncoding oligos. TtAgo uses 5′-phosphorylated 13-25nt DNA as guides in archaeal host defence via DNA interference (DNAi) [[1](#_ENREF_1)]. We made use of the same plasmids for TtAgo expression, which have been made available by those authors (Addgene), i.e. pWUR702 (TtAgo) and pWUR703 (loss-of-function TtAgo [D478A, D546A]). Using the PCNA1-template assay system and *Taq* polymerase as described above for qPCR, we replaced 27nt-RNA with 5’-phosphorylated 21nt-DNA or analogous 21nt-ssRNA. The qPCR results demonstrate that 21nt-DNA or 21nt-ssRNA alone possesses the potential to impair DNA amplification (Figure 5B). This effect is massively strengthened between 10mM and 400nM oligo concentration, when TtAgo is added in a 1:2 (TtAgo:21nt-DNA) molar ratio. However, guide DNA loaded Ago can cleave target DNA in a temperature dependent manner, whereas TtAgo:21nt-ssRNA complexes do not exhibit cleavage activity [[1](#_ENREF_1)]. At 10mM 21nt-ssRNA concentration TtAgo the inhibitory effect was stronger than with oligonucleotides alone. Adding non-functional TtAgo reverted the observed effects leading to amplification efficiencies similarly to conditions, when purely 21nt-DNA or 21nt-ssRNA was used. The results indicate that the presence of an Argonaute family protein in TtAgo:21nt-RNA complexes can strengthen the DNA replication-interference through oligonucleotides, whereby most inhibitory activity must be assigned to the target DNA cleavage activity, when TtAgo:guide DNA was used. Nevertheless, the latter observation indicates that these complexes target complementary DNA properly.


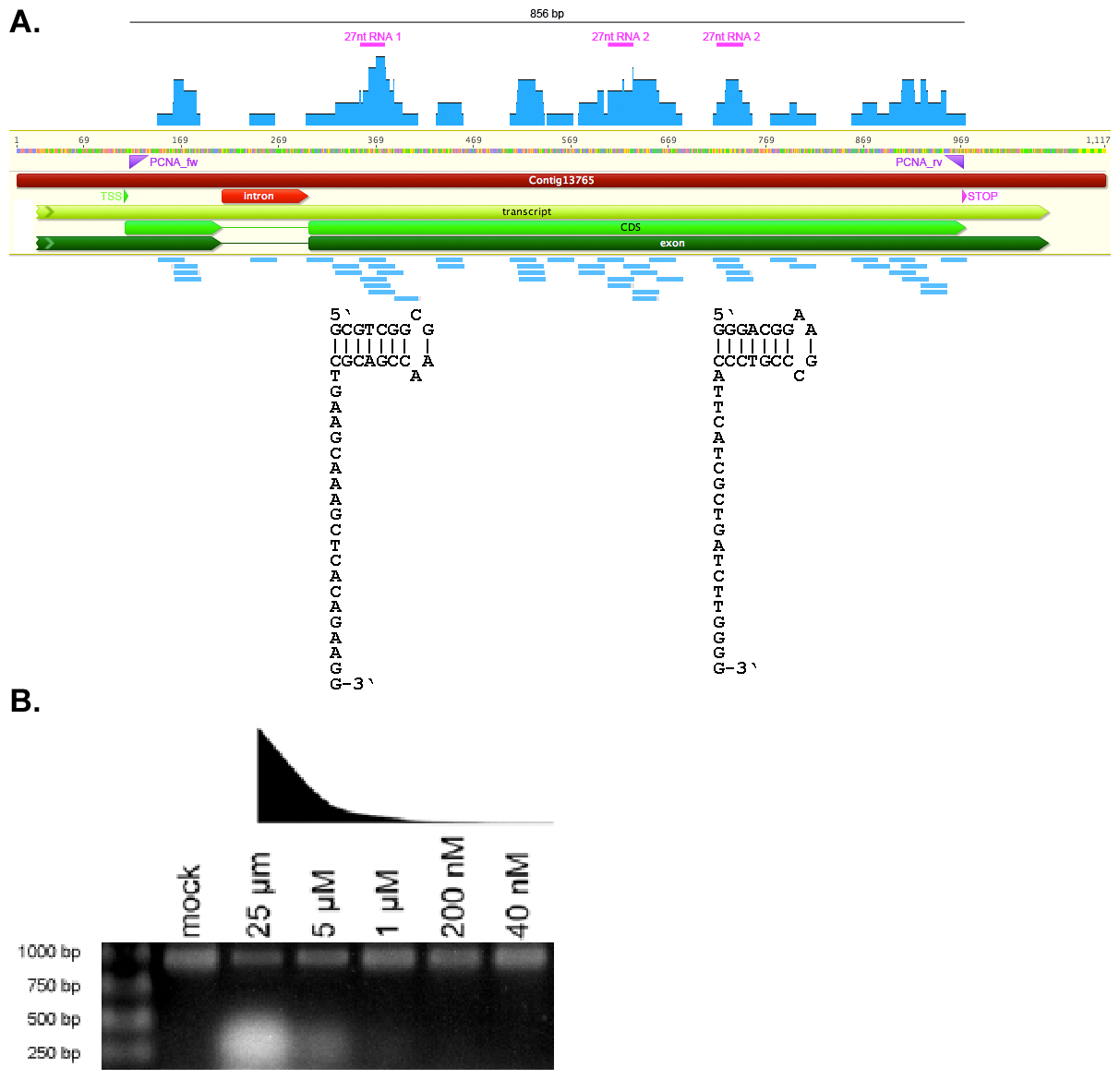


**Figure S6. The PCNA1 nanochromosome and its use for replication interference assays.** A template for PCR and *Klenow* reaction assays was constructed by cloning a 856bp PCR fragment from the PCNA nanochromosome into pGEM-T-easy (Promega). The PCNA fragment harbours binding sites for three 27nt-RNAs (or 5’-phosphorylated 21nt-DNA overlapping) used in in vitro assays, which are indicated. Below is the 2ndary structure of PCNA primer construct, whose 3’-end specifically bind PCNA as indicated, but whose 5’-overlength regions build up hairpin-loop structures to minimize binding by Argonaute proteins.


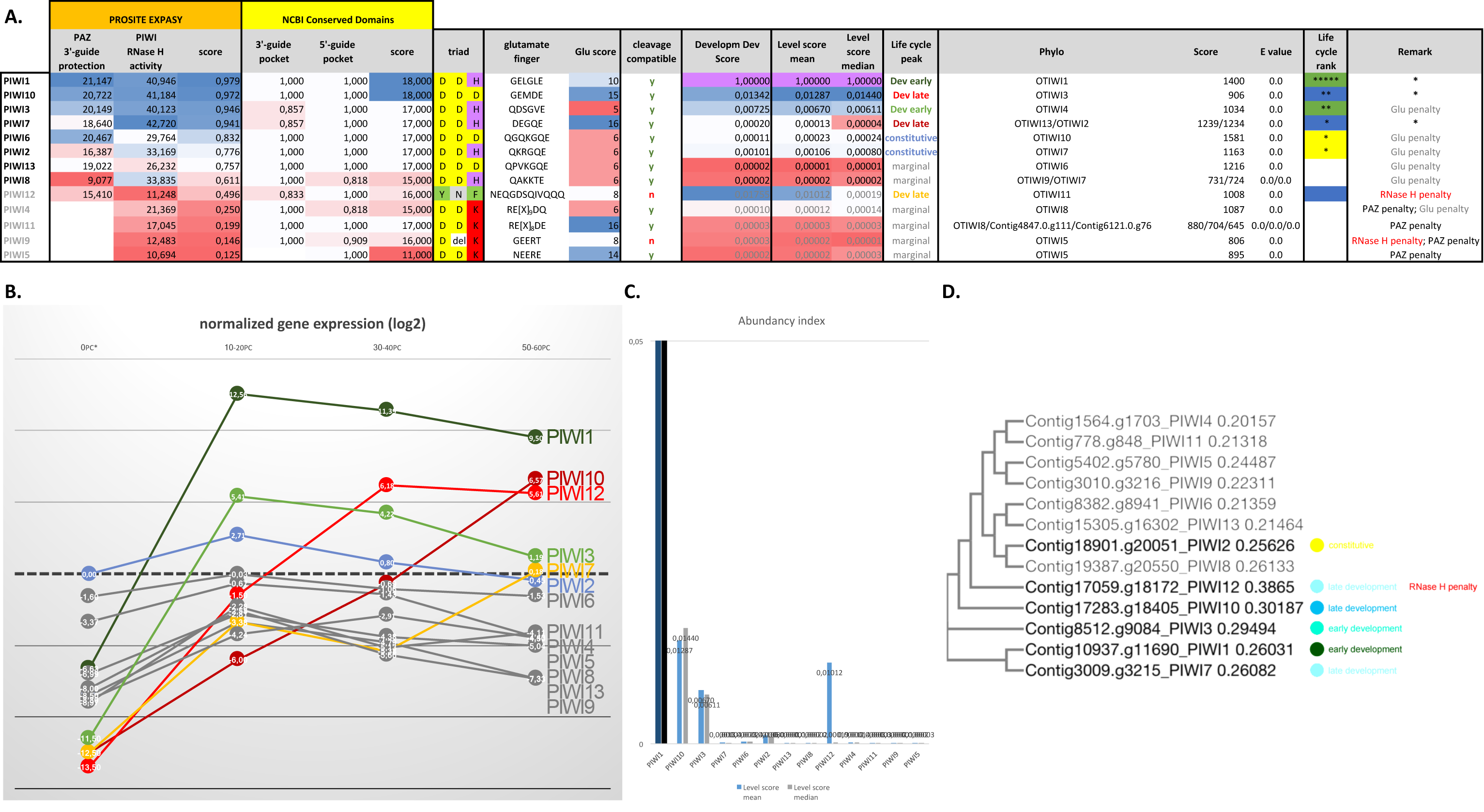


**Figure S7. Features and considerations about *Stylonychia* PIWI-domain containing proteins and comparison with *Oxytricha* OTIWIs.** A. The table gives an overview about several PIWI-domain features, such as PAZ and PIWI domain conservation scores (calculated with either Prosite [http://prosite.expasy.org/] or NCBI conserved domain search [https://www.ncbi.nlm.nih.gov/Structure/cdd/wrpsb.cgi]) and information about the presence of a DDH triad and a glutamate finger in the RNase H domain, which would be reminiscent of slicing Argonautes and allows to speculate about the cleavage compatibility of a given PIWI (‘y’/yes or ‘n’/no). Moreover, scores for each PIWI’s abundance during macronuclear differentiation were calculated from the relative PIWI mRNA levels during development vs. vegetative growth, and the *Oxytricha* homologs were identified for each *Stylonychia* PIWI. Notably, PIWI2 becomes constitutively expressed during vegetative growth and sexual reproduction, whereas its abundance during macronuclear development is several magnitudes lower than PIWI1 (See graphical illustration of PIWI’s gene expression [B.] and their relative abundance index for exconjugant cells 20h PC [C.], where PIWI1 [index=1.0] was used as reference. The y-axis was cut at 0.05 for the sake of readability.). D. The phylogenetic relationship between *Styloynchia* PIWI’s was analysed with the Neighbor-joining option (Bootstrap test; 1000 replicates) using MEGA7 software [[2](#_ENREF_2)].

| **Time point** | **t_1_** | **t_2_** | **t_3_** |
| --- | --- | --- | --- |
| **Condition** | **MDS protection:**  MDS replication stalled | **Release of MDS protection:**  MDS replication ongoing | **Polytene chromosome formation terminated:**  ⇒ resulting signature |
| **Availability of**  **H3.5\|H3(.4)K27me3** | **+\|-**  Assembly of H3.5 to **bulk DNA** | **+\|-**  Assembly of H3.5 to **MDSs** | No H3.4K27me3 would occur; or bulk DNA-specific targeting mechanism for H3K27-specific histone KMT would be required.  **⇒ most probably invalid.** |
|  | **+\|+**  Assembly of H3.5 and H3.4K27me3 to **bulk DNA** | **+\|+**  Assembly of **H3.5** and H3.4K27me3 to **MDSs** | Non-differential distribution of H3.4K27me3 would be expected; or sequence-specific targeting and selective assembly of histone variants would be required.  **⇒ most probably invalid.** |
|  | **+\|-**  Assembly of H3.5 to **bulk DNA** | **+\|+**  Assembly of H3.5 and H3.4K27me3 to **MDSs** | MDSs would be assembled with H3.4K27me3 and H3.5.  **⇒ invalid.** |
|  | **+\|+**  Assembly of H3.5 and H3.4K27me3 to **bulk DNA** | **+\|-**  Primarily assembly of H3.5 to **MDSs** | Preferentially bulk DNA would be enriched with H3.4K27me3 and with H3.5, whereas MDSs would be associated (mainly) with H3.5.  **⇒ valid scenario.** |

**Table S2.** Theoretical scenarios and consequences of differential histone variant occurrence under the conditions of RIRI.

**Supplemental references**

1. Swarts DC, Jore MM, Westra ER, Zhu Y, Janssen JH, Snijders AP, Wang Y, Patel DJ, Berenguer J, Brouns SJ *et al*: **DNA-guided DNA interference by a prokaryotic Argonaute**. *Nature* 2014, **507**(7491):258-261.

2. Kumar S, Stecher G, Tamura K: **MEGA7: Molecular Evolutionary Genetics Analysis Version 7.0 for Bigger Datasets**. *Molecular biology and evolution* 2016, **33**(7):1870-1874.
